# Dual functions of *PsmiR172b-PsTOE3* module in dormancy release and flowering in tree peony (*Paeonia suffruticosa*)

**DOI:** 10.1093/hr/uhad033

**Published:** 2023-02-21

**Authors:** Yuxi Zhang, Linqiang Gao, Yanyan Wang, Demei Niu, Yanchao Yuan, Chunying Liu, Xinmei Zhan, Shupeng Gai

**Affiliations:** College of Life Sciences, Qingdao Agricultural University, Qingdao, 266109, China; University Key Laboratory of Plant Biotechnology in Shandong Province, Qingdao, 266109, China; College of Life Sciences, Qingdao Agricultural University, Qingdao, 266109, China; University Key Laboratory of Plant Biotechnology in Shandong Province, Qingdao, 266109, China; College of Life Sciences, Qingdao Agricultural University, Qingdao, 266109, China; University Key Laboratory of Plant Biotechnology in Shandong Province, Qingdao, 266109, China; College of Life Sciences, Qingdao Agricultural University, Qingdao, 266109, China; University Key Laboratory of Plant Biotechnology in Shandong Province, Qingdao, 266109, China; College of Life Sciences, Qingdao Agricultural University, Qingdao, 266109, China; University Key Laboratory of Plant Biotechnology in Shandong Province, Qingdao, 266109, China; College of Life Sciences, Qingdao Agricultural University, Qingdao, 266109, China; University Key Laboratory of Plant Biotechnology in Shandong Province, Qingdao, 266109, China; College of Life Sciences, Qingdao Agricultural University, Qingdao, 266109, China; University Key Laboratory of Plant Biotechnology in Shandong Province, Qingdao, 266109, China; College of Life Sciences, Qingdao Agricultural University, Qingdao, 266109, China; University Key Laboratory of Plant Biotechnology in Shandong Province, Qingdao, 266109, China

## Abstract

MicroRNAs (miRNAs) are non-coding RNAs that interact with target genes and are involved in many physiological processes in plants. miR172-AP2 mainly plays a role in the regulation of flowering time and floral organ differentiation. Bud dormancy release is necessary for forcing culture of tree peony in winter, but the mechanism of dormancy regulation is unclear. In this study, we found that a *miR172* family member, *PsmiR172b*, was downregulated during chilling-induced bud dormancy release in tree peony, exhibiting a trend opposite to that of *PsTOE3*. RNA ligase-mediated (RLM) 5′-RACE (rapid amplification of cDNA ends) confirmed that *miR172b* targeted *PsTOE3*, and the cleavage site was between bases 12 (T) and 13 (C) within the complementary site to *miR172b*. The functions of *miR172b* and *PsTOE3* were detected by virus-induced gene silencing (VIGS) and their overexpression in tree peony buds. *PsmiR172b* negatively regulated bud dormancy release, but *PsTOE3* promoted bud dormancy release, and the genes associated with bud dormancy release, including *PsEBB1*, *PsEBB3*, *PsCYCD*, and *PsBG6*, were upregulated. Further analysis indicated that PsTOE3 directly regulated *PsEBB1* by binding to its promoter, and the specific binding site was a C-repeat (ACCGAC). Ectopic expression in *Arabidopsis* revealed that the *PsmiR172b*-*PsTOE3* module displayed conservative function in regulating flowering. In conclusion, our results provided a novel insight into the functions of *PsmiR172-PsTOE3* and possible molecular mechanism underlying bud dormancy release in tree peony.

## Introduction

MicroRNAs (miRNAs) are non-coding RNAs with a length of 20–24 nucleotides. They are involved in many development processes in plants. Some plant miRNAs regulate the associated gene expression by targeting the encoding region rather than the 3′-untranslated regions (UTRs) at post-transcription level [1–3]. miR156 and miR172, targeting *SQUAMOSA PROMOTER BINDING PROTEIN-LIKE* (*SPL*) and *APETALA2* (*AP2* or *AP2*-like), respectively, coregulate vegetative transition, seed dormancy, flowering time, and stress responses, etc. [4–9]. The miR172 family is highly conserved across the plant kingdom; it was first discovered in *Arabidopsis* and includes five members, *miR172a*–*e* [10]. Sakuma *et al*. characterized 144 members of the AP2 family based on the number and sequence of the AP2 domain, and divided them into five subfamilies, comprising AP2, ERF, DREB, RAV, and Soloists in *Arabidopsis* [11]. Among them, AP2 subfamily members are mainly involved in plant development. The *Arabidopsis* AP2 subfamily includes AP2, TARGET OF EAT (TOE1, TOE2, and TOE3), SCHLAFMUTZE (SMZ), and SCHNARCHZAPFEN (SNZ). Among them, the AP2 subgroup has two conserved AP2 domains with a YRG motif at the N-terminus and an RAYD motif at the C-terminus [12]. Every subfamily of the AP2 family has a specific DNA-binding motif. For example, ethylene-responsive transcription factor (ERF) can bind to the ethylene response element GCC box [13]; AINTEGUMENTA (ANT) can specifically bind to 5′-gCAC(A/G)N(A/T)TcCC(a/g)ANG(c/t)-3′, and the binding site for the AP2/EREBP subfamily contains the C-repeat (ACCGAC) [14].

Studies on *miR172* and its target genes mainly focus on the regulation of flowering time and floral organ differentiation. In *Arabidopsis*, six *AP2*-like genes (*AP2*, *TOE1*, *TOE2*, *TOE3*, *SMZ*, and *SNZ*) are regulated by five *miR172* family members (*miR172a*–*e*). *miR172* overexpression results in early flowering, and the mutants, including *toe1toe2*, *toe1toe2smzsnz*, and *toe1toe2toe3smzsnzap2*, exhibit precocious flowering [15]. The overexpression of *miR172a* in *Gloxinia* accelerates flowering by repressing *SsAP2-like*; however, no obvious changes are observed in the flowers [16].

It is reported that common signaling intermediates between flowering time and endodormancy regulation in trees [17]. Bud dormancy of perennial woody plants is an adaptive strategy for survival under unfavorable conditions [18]. Recently, several AP2-type transcription factors have been reported to be involved in bud dormancy release. In poplar, EARLY BUD-BREAK 1 (EBB1), a putative AP2/ERF protein, was first identified to accelerate the seasonal dormancy release [19]. Further, *EBB3* was identified, whose upregulation activates the transcription of *CYCD3.1* to promote budbreak [20]. Expression profiling of small RNAs and mRNA revealed that *PagmiR172* targeting *AP2* is differentially expressed from endodormancy to the active growth stage in poplar [21]. In addition, poplar *EBB1* homology genes in other woody perennial plants, including *Prunus persica*, *Malus domestica*, *Pyrus pyrifolia* and *Vitis vinifera*, have a conserved role as positive regulators of budbreak [22]. The C-repeat binding factors (CBFs), including CBF1, CBF3, CBF4, and CBF5, belonging to the AP2 family and CBF/DREB subfamily, can bind to the promoter of *PmDAM6* (dormancy associated MADS-box) to affect bud dormancy in *Prunus mume* by forming alternative protein complexes [23]. Therefore, AP2 and miR172 family members might play a vital role during dormancy release in perennial woody plants.

Tree peony (*Paeonia suffruticosa* Andrews), which originated in China, is a famous ornamental plant, and its bud dormancy is endodormancy [24]. Forcing cultivation for the Spring Festival in China has become essential for the tree peony industry. However, the main limiting factor for bud burst and flower quality is the complete release of bud endodormancy. Therefore, it is very urgent to elucidate the mechanism underlying bud dormancy release in tree peony. Measures such as application of artificial chilling, exogenous gibberellins (GAs) [25], garlic paste [26], and 5-azacytidine (5-azaC) [27] have been taken for dormancy release. Among them, sufficient application of low temperature is an effective and common way to break bud dormancy in tree peony. In the last two decades, many studies have focused on elucidating the molecular mechanism of bud dormancy in tree peony. Several differentially expressed unigenes associated with dormancy release have been screened using suppression subtractive hybridization (SSH) and cDNA microarrays [28]. Cell division is gradually reinitiated and accelerated at the end of endodormancy [29]. The corresponding cyclin gene, *PsCYCD*, is upregulated, and acts at the G1–S transition [28]. At the same time, bud dormancy release is accompanied by the reopening of transport channels, and the β-1,3-glucanase gene *PsBG6* plays a vital role in this process [30]. Additionally, an AP2 member is dramatically upregulated after sufficient exposure to chilling at both transcription and translation level [31]. *PsmiR172* family members are differentially expressed during chilling-induced dormancy release in tree peony ‘Fendanbai’, and *AP2-like* is predicted as its target gene [32]. However, the *miR172* member that plays a major role in peony bud dormancy release and the intrinsic molecular mechanism is still unclear.

Here, we identified *miR172* family members involved in bud dormancy release in tree peony ‘Luhehong’ and observed that *miR172b* targeted *PsTOE3* by cleaving its transcript. The functions of *PsmiR172b* and *PsTOE3* were detected using virus-induced gene silencing (VIGS) and their overexpression. Interestingly, PsTOE3 could bind to the promoter of *PsEBB1* to regulate its transcription, which might finally accelerate cell division and promote bud dormancy release.

## Results

### Expression patterns of *PsmiR172s* family members during chilling-induced bud dormancy release

**Figure 1:**
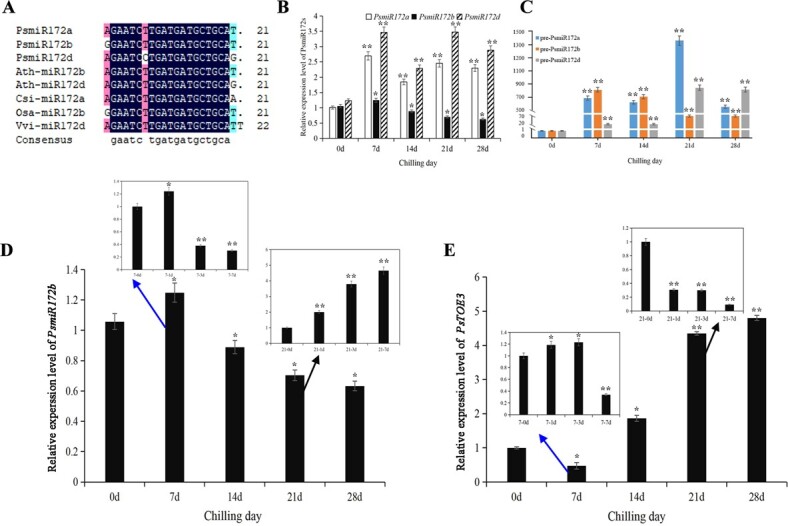
Homology comparison of *miR172s* in tree peony and the other plants, and expression patterns of *PsmiR172s* and *PsTOE3* during chilling-induced bud dormancy release. (A) Homology comparison of *miR172s* in tree peony and the other plants. Ps, *Paeonia suffruticosa*; Ath, *Arabidopsis thaliana*; Csi, *Camellia sinensis*; Osa, *Oryza sativa*; Vvi, *Vitis vinifera*. (B) Expression levels of mature *PsmiR172a*, *PsmiR172b*, and *PsmiR172d* during chilling-induced dormancy release in tree peony. (C) Expression levels of pre-*PsmiR172a*, pre-*PsmiR172b*, and pre-*PsmiR172d* during chilling-induced dormancy release in tree peony. (D and E) Expression levels of *PsmiR172b* and *PsTOE3* after chilling and after being transferred to the greenhouse when exposed to chilling for 7 and 21 days. Blue arrows show the relative expression levels of *PsmiR172b* and *PsTOE3* when being transferred to the greenhouse after chilling for 7 days, and black arrows show the relative expression levels of *PsmiR172b* and *PsTOE3* when transferred to the greenhouse after chilling for 21 days. Data are the mean ± standard deviation of three replications. ^*^Significant difference at *P* < .05, ^**^significant difference at *P* < .01.

Based on our recent miRNA sequencing [32], three *PsmiR172* members were obtained after comparison with other *miR172* family members in miRBase, which were named *PsmiR172a*, *PsmiR172b*, and *PsmiR172d* (Fig. 1A). The expression levels of three *PsmiR172s* were determined during the chilling-induced bud dormancy process using real-time quantitative RT–PCR (qRT–PCR) (Fig. 1B). The results revealed that the expression levels of mature *PsmiR172a* and *PsmiR172d* were significantly upregulated after chilling for 7 days, and the expression was maintained at relatively high levels with the prolongation of chilling treatment until 28 days. The expression of *PsmiR17b* was slightly increased after 7 days of chilling treatment, followed by a continuous decrease until 28 days of chilling (Fig. 1B). The mature *PsmiR172s* sequences were used to perform BLAST against the draft peony genome sequence for identification of the three precursor sequences [33]. The precursors of *PsmiR172s* were amplified from the genomic DNA of ‘Luhehong’. The sequences are given in Supplementary Data File 1. Their foldback structures were predicted using TBTools; they could form characteristic stem–loop structures (Supplementary Data Fig. S1). Therefore, these three sequences were designated as *pre-miR172a*, *pre-miR172b*, and *pre-miR172d*. The expression patterns of these precursors during chilling-induced dormancy release were determined using qRT–PCR. The results indicated that *pre-miR172a* and *pre-miR172d* were upregulated, and their levels reached their peak at 21 days of chilling. The expression level of *pre-miR172b* was maximal at 7 days of chilling, followed by a decrease (Fig. 1C).

When plants that had been chilled for 7 days were transferred to a greenhouse, the expression level of *PsmiR172b* significantly increased after 1 day, followed by a significant decrease until 7 days. After 21 days of chilling followed by transfer of the plants into a greenhouse, *PsmiR172b* was significantly induced at 22°C after 1 day and was steadily upregulated until 7 days (Fig. 1D). These results indicated that the buds of dormancy release could sustain high expression of *PsmiR172b* after being moved to growth conditions.

### Cloning and expression analysis of *PsTOE3* during peony bud dormancy release

**Figure 2:**
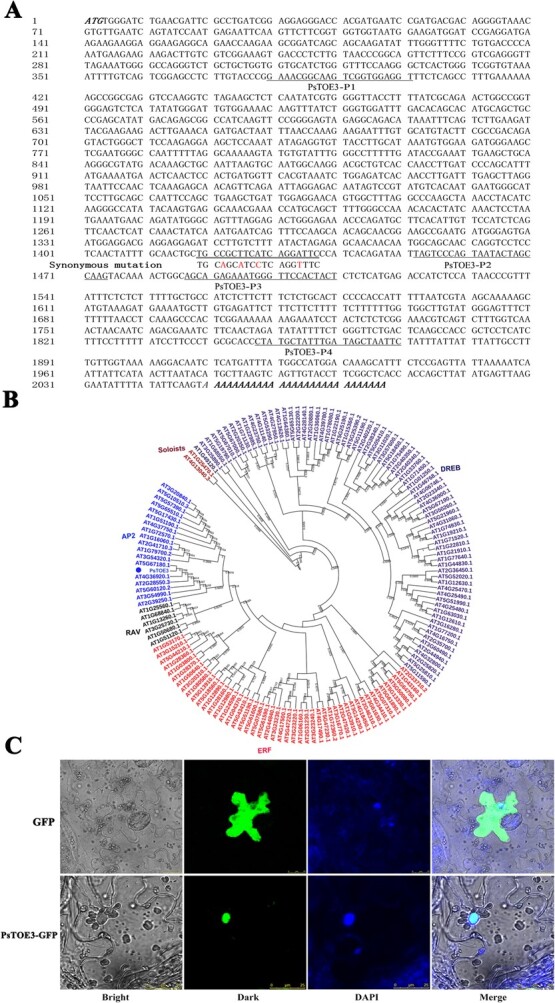
cDNA sequence of *PsTOE3*, phylogenetic tree, and subcellular localization of PsTOE3. (A) cDNA sequence of *PsTOE3*. The initiation codon and termination codon are marked by bold italics, and the putative binding sites of *PsmiR172b* are marked by double underline; the synonymous mutation sites (for mPsTOE3) are marked by a red color. The primer (PsTOE3-P1, -P2, -P3, and -P4) sites for RT–PCR are marked by underlines. (B) Phylogenetic tree containing PsTOE3 and 147 AtAP2/ERFs constructed using the neighbor-joining method with 1000 bootstrap replications. Different colors mark different subfamilies of the AP2 family, and PsTOE3 is marked with a blue dot. (C) Subcellular localization of PsTOE3 by fluorescent microscopy with a stimulating wavelength of 488 nm.

A total of 173 AP2-like family members were obtained from the *P. suffruticosa* genome by local BLAST [33]. Among them, there were 14 AP2-like subfamily members, and only 3 AP2-like subfamily members were expressed in buds during chilling-induced dormancy release [28]. The expression levels of three AP2-like subfamily members were determined using qRT–PCR. Contig 18772 (GenBank accession number JI446524) was slightly inhibited after 7 days of chilling, followed by continuous upregulation for up to 28 days of chilling (Fig. 1E). Expression of the other contigs (GenBank accession numbers JI447049 and JI458458) was induced after chilling for 7 days, followed by continuous downregulation (Supplementary Data Fig. S2). After target gene prediction, only the contig with accession number JI446524 had the complementary site of *PsmiR172b* in the encoding frame. Rapid amplification of cDNA ends (RACE)–PCR was performed to obtain its full cDNA sequence, and 750-bp 5′-RACE fragment and 1500-bp 3′-RACE fragments were amplified. After splicing and assembling, a 2159-bp cDNA sequence was obtained, comprising a 1533-bp coding region (Fig. 2A), an 82-bp 5′-UTR, and a 544-bp 3′-UTR (GenBank accession number KR608302).

The open reading frame (ORF) encoded 510 amino acids with a calculated molecular mass of 56.925 kDa and predicted pI of 6.66; it contained two conserved DNA-binding domains identified as AP2 domains (151–206 and 242–302). WESH (164–167) and WEAR (255–258) motifs were found in the two AP2 domains. The subcellular localization prediction revealed that it might be located in the nucleus. The phylogenetic tree was constructed containing the putative AP2 protein and 147 AP2 family proteins in *Arabidopsis.* The putative protein was clustered into the AP2 subfamily, which was first clustered with AtTOE3 (AT5G67180) and shared 70.45% sequence identity (Fig. 2B). Therefore, it was named *PsTOE3* according to the closest *Arabidopsis* homolog [34]*.*

The expression levels of *PsTOE3* during chilling-induced bud dormancy release suggested that *PsTOE3* might promote bud dormancy release. After chilling for 7 days followed by transfer to growth conditions, *PsTOE3* was significantly induced after 1 day of indoor-temperature treatment, and maintained a high expression level until 3 days (Fig. 1E). However, the transcript levels were dramatically downregulated for buds after chilling for 21 days. We assumed that the regrowth of buds might not require high expression of *PsTOE3*.

The fusion expression vector 35S::*PsTOE3:GFP* was constructed and transformed into tobacco leaves, with 35S::GFP as the control. PsTOE3 was located in the nucleus as revealed by DAPI (4′,6-diamidino-2-phenylindole) staining and laser scanning confocal microscopy observation (Fig. 2C).

### *PsmiR172b* targets *PsTOE3*

**Figure 3:**
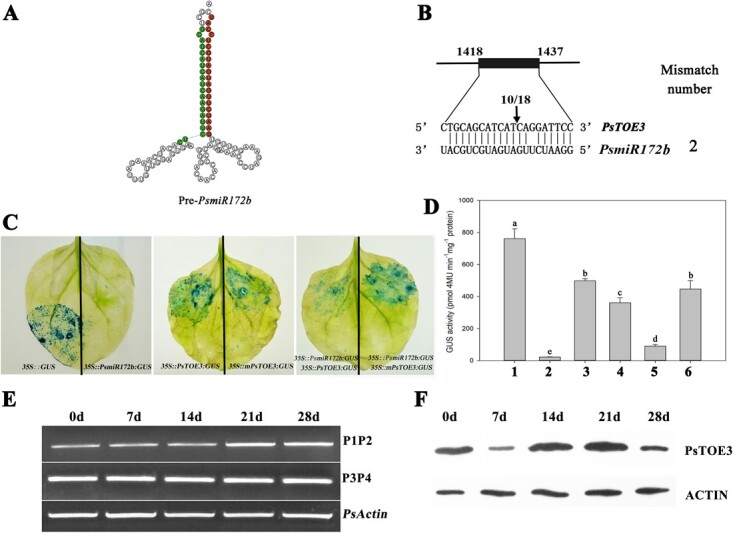
Prediction and validation of the target relationship between *PsTOE3* and *PsmiR172b*. (A) Secondary structure of *PsmiR172b* precursor. Red represents the sequence of mature *PsmiR172b* and its complementary sequence is marked in green. (B) Identification of the *PsmiR172b* target site with RLM 5′-RACE. The cleaved site is marked with an arrow. The numbers above the arrow are the numbers of cleavage sites in independent clones. (C) Cotransformation of tobacco leaves with *PsmiR172b:GUS* and *PsTOE3:GUS*, *PsmiR172b:GUS*, and *mPsTOE3:GUS*, respectively*.* GUS staining was observed histochemically. (D) Quantitative analysis of GUS enzyme activities by fluorospectrophotometer in leaves inoculated with different recombinant vectors. 1, 35S::GUS; 2, 35S::*PsmiR172b:GUS*; 3, 35S::*PsTOE3:GUS*; 4, 35S::*mPsTOE3:GUS*; 5, 35S:: *PsmiR172b:GUS* + 35S::*PsTOE3:GUS*; 6, 35S::*PsmiR172b:GUS* + 35S::*mPsTOE3:GUS*. Data are the mean ± standard error of the mean of 10 plants. Lowercase letters on the columns indicate significant difference at the *P* < .05 level. (E) Change trends of *PsTOE3* transcripts during chilling-induced dormancy release using RT–PCR. Primer pair P1 and P2 was used to amplify the fragment containing the target region, and P3 and P4 were used to obtain the fragment after the target region. The primer sites are showed in Fig. 2A. (F) Protein levels of PsTOE3 during chilling-induced dormancy release by western blot; ACTIN protein was the internal control.

The opposite expression patterns of *PsmiR172b* and *PsTOE3* during chilling-induced dormancy release suggested that *PsmiR172b* might target *PsTOE3* and regulate its expression (Fig. 1D and E). The secondary hairpin structure of 99-bp *prePsmiR172b* could construct the characteristic stem-loop structure, and the mature *PsmiR172* located on the 3′ arm of *pre-PsmiR172b* (Fig. 3A). In addition, target gene prediction using RNAhybrid software supported *PsmiR172b* targeting *PsTOE3*, and the complementary site of *PsmiR172b* was located at 1418–1437 bp of the *PsTOE3* ORF (Fig. 3B).

To confirm the target relationship between them, the 230-bp *PsmiR172b* precursor was used to construct the fusion expression vector *PsmiR172b:GUS*. The target sites of *PsmiR172b* were replaced with the synonymous mutation bases to generate *mPsTOE3* as the negative control (Fig. 2A). *PsTOE3:GUS* and *mPsTOE3:GUS* fusion expression vectors were constructed, and used to transform tobacco leaves with *PsmiR172b:GUS*. No GUS signal was detected in the leaves inoculated with *PsmiR172b:GUS* alone; however, strong GUS (β-glucuronidase) signals were detected when *mPsTOE3:GUS* alone was introduced into tobacco or cotransformed with *PsmiR172b:GUS*. Compared with the control (*mPsTOE3:GUS* and *PsmiR172b:GUS*), cotransformation using *PsTOE3:GUS* and *PsmiR172b:GUS* exhibited a significant suppression of the GUS signal (Fig. 3C). GUS activities were consistent with the GUS staining results (Fig. 3D), confirming the target relationship between *PsmiR172b* and *PsTOE3*.

Further, we investigated whether *PsmiR172b* regulated *PsTOE3* at the post-transcription or -translation level. Based on the target sites of *PsmiR172b* on *PsTOE3*, the primer pairs P1 and P2 were used to amplify the fragment containing the target region, and P3 and P4 were used to obtain the fragment after the target region. The amplification product of P1 and P2 exhibited an upward trend during chilling-induced dormancy release, whereas no significant change was observed with that of P3 and P4 (Fig. 3E). This was basically consistent with the qRT–PCR result of *PsTOE3* during chilling-induced dormancy release (Fig. 1D).

To confirm whether *PsmiR172* regulated *PsTOE3* at the post-transcription or -translation level, MBP-PsTOE3 recombinant vector was constructed and anti-PsTOE3 antibody was prepared. Western blot analysis indicated that the PsTOE3 protein level was decreased after 7 days of chilling, followed by an increase after 14 days of chilling, and it maintained its high levels until 21 days of chilling (Fig. 3E), which was consistent with the change in the *PsTOE3* transcripts (Fig. 1D). To further elucidate the splicing site, RNA ligase-mediated (RLM) 5′-RACE was performed. The cleaved site was between bases 12 (T) and 13 (C) within the complementary site to *PsmiR172b* (Fig. 3B). Because miRNA regulates its target genes by transcription RNA shear or translation inhibition, our results confirmed a post-transcriptional regulation of *PsTOE3* by *PsmiR172b* during bud dormancy release, which led to the variation in the PsTOE3 protein quantity.

### Silencing *PsmiR172b* and overexpression of *PsTOE3* promote dormancy release

**Figure 4:**
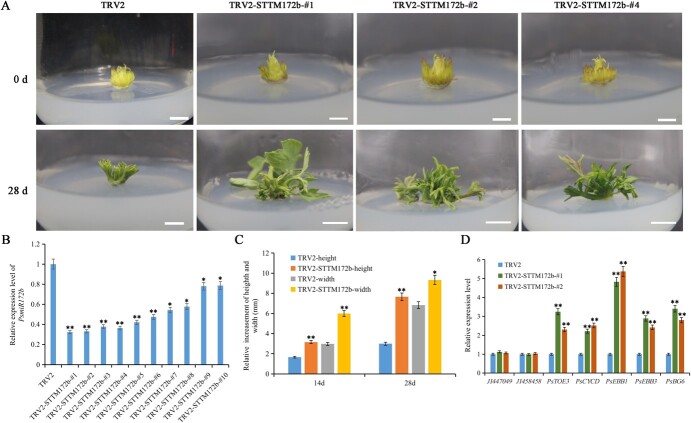
Morphological changes and expression levels of genes associated with bud dormancy release after silencing of *PsmiR172b* by VIGS in tree peony. (A) Morphological changes after silencing of *PsmiR172d* by VIGS for 28 days. Buds infected with TRV1/TRV2 were used as control. Bar = 1.0 cm. (B) Relative expression levels of *PsmiR172b* in buds after silencing of *PsmiR172b* for 7 days. (C) Relative growth of height and width of TRV2-*STTM172b* buds after transforming for 14 and 28 days. (D) Relative expression levels of genes associated with peony dormancy release in TRV2-*STTM172b* buds after transforming for 7 days. Data are the mean ± standard deviation of six replications for relative expression. ^*^Significant difference at *P* < .05, ^**^significant difference at *P* < .01.

To confirm the function of *PsmiR172b*, *PsmiR172b* was knocked down using a tobacco rattle virus (TRV)-mediated short tandem target mimic (STTM) approach. The TRV2*-STTM172b* vector was constructed and used to transform tree peony buds of 7 days of chilling. Seven days after transformation, the expression levels of *PsmiR172b* in 10 transformed buds were significantly suppressed with silencing efficiency of 67.7, 66.9, 62.2, 63.5, 57.9, 52.4, 45.7, 42.2, 22.1, and 21.4%, respectively (Fig. 4B). After 14 days, the *PsmiR172b*-silenced buds burst faster and were taller and wider, which is an important index of bud dormancy release (Fig. 4C). After 28 days, significant morphological changes were observed in *PsmiR172b-*silenced buds compared with TRV2-vector-transformed buds (control); the relative growths of height and width in TRV2*-PsmiR172b* buds were higher than those in the control (Fig. 4A and C). Two buds (TRV2-*STTM172b*-#1 and TRV2-*STTM172b*-#2) with silencing efficiency of 67.7 and 66.9%, respectively, were used for further analysis. The target gene, *PsTOE3*, was significantly upregulated in these two buds, and the expression levels of *PsEBB1*, *PsEBB3*, *PsCYCD*, and *PsBG6* were higher than those in the control (Fig. 4D). In addition, the expression levels of the other AP2-like subfamily members in *PsmiR172b-*silenced buds were not significantly different (Fig. 4D), which might be because there were no target sites of *PsmiR172b* in their mRNA sequences. Therefore, the silencing of *PsmiR172b* promoted tree peony bud dormancy release and budburst in tree peony.

**Figure 5:**
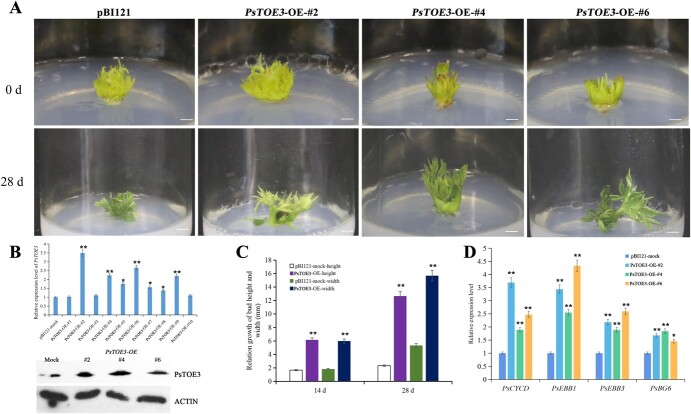
Morphological changes and expression levels of genes associated with bud dormancy release after overexpression of *PsTOE3* in tree peony. (A) Morphological changes in *PsTOE3* overexpression buds after transformation for 28 days. Buds with empty pBI121 vector were used as control. Scale bar = 1.0 cm. (B) Relative expression levels of *PsTOE3* in *PsTOE3*-OE buds using qRT–PCR and the amount of PsTOE3 protein in three *PsTOE3*-OE buds (#2, #4, and #6) using western blot after transformation for 7 days. (C) Relative growth in height and width in *PsTOE3*-OE buds after transformation for 14 and 28 days. (D) Relative expression levels of genes associated with peony dormancy release using qRT–PCR in *PsTOE3*-OE buds after transformation for 7 days. Data are the mean ± standard deviation of six replications for relative expression. ^*^Significant difference at *P* < .05, ^**^significant difference at *P* < .01.

In addition, the overexpression vector pBI121*-PsTOE3* was constructed and used to transform *Agrobacterium tumefaciens* strain EHA105, which infected peony buds. After 7 days, 10 buds were randomly selected to assess the expression of *PsTOE3*, which was higher in seven of them than in buds with empty pBI121 vector (control). The level of PsTOE3 proteins in three *PsTOE3*-OE buds (#2, #4, and #6) significantly increased (Fig. 5B). After 14 days of transformation, the relative growths in height and width of buds with *PsTOE3* overexpression (*PsTOE3-*OE) were higher than those of control buds, and the changes in the height and width were more significant after 28 days (Fig. 5A and C). The expression levels of *PsEBB1*, *PsEBB3*, *PsCYCD*, and *PsBG6* in three *PsTOE3*-OE buds (#2, #4, and #6) were significantly increased; the expressions of *PsEBB1*, *PsEBB3*, and *PsCYCD* in *PsTOE3*-OE-#6 increased ~4.33, 2.59, and 2.47 times, respectively (Fig. 5D). Altogether, the analysis of morphological changes and the expression of genes associated with dormancy release of *PsTOE3*-OE buds confirmed that *PsTOE3* promoted bud dormancy release.

### PsTOE3 directly promotes *PsEBB3* expression

**Figure 6:**
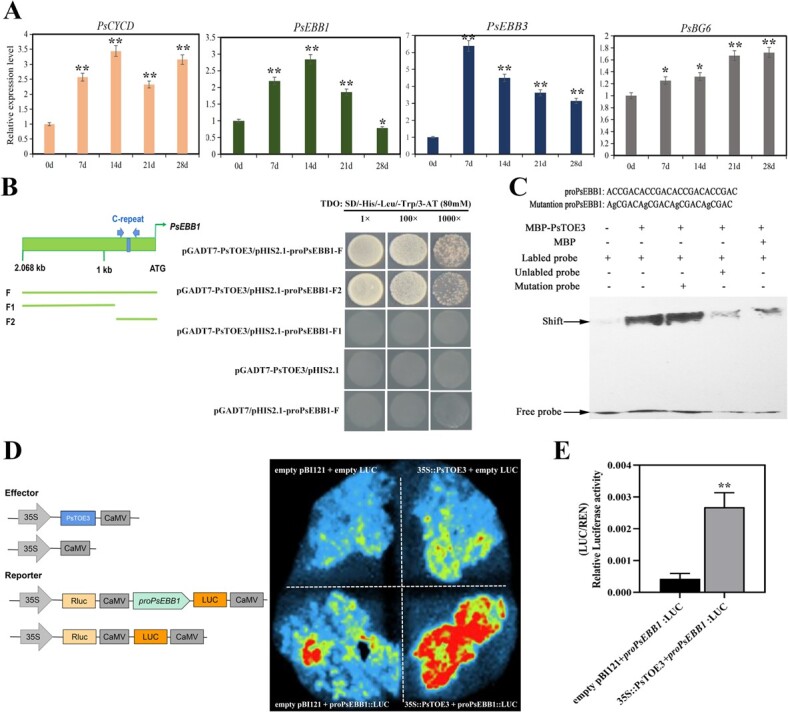
Transcript levels of the genes associated with dormancy release as revealed by qRT–PCR and PsTOE3 directly regulated the expression of *PsEBB1*. (A) Expression levels of the genes associated with dormancy release, including *PsCYCD*, *PsEBB1*, *PsEBB3*, and *PsBG6* during chilling-induced dormancy release using qRT–PCR. (B) Schematic diagram of *PsEBB1* promoter and the result of the yeast one-hybrid assay. The C-repeat is marked with blue arrows. (F) Full length of *PsEBB1* promoter; F1 (−2068 to −629), a fragment without C-repeat as a negative control; F2 (−628 to −1), a fragment with C-repeat. (C) Interaction of PsTOE3 protein and the C-repeat promoter of *PsEBB1* by EMSA. The C-repeat (ACCGAC) and mutation C-repeat (AGCGAC) were synthesized with four replications, and the mutation C-repeat was used as negative control. (D) Regulation of PsTOE3 protein to *PsEBB1* using the dual luciferase assay after infiltration for 3 days. (E) Relative LUC/REN activities after infiltration for 3 days. Data are the mean ± standard deviation of six replications for relative expression. ^*^Significant difference at *P* < .05, ^**^significant difference at *P* < .01.

The expressions of *PsCYCD*, *PsEBB1*, *PsEBB3*, and *PsBG6* after silencing *PsmiR172b* and overexpression of *PsTOE3* were significantly increased. Further, we assessed whether *PsCYCD*, *PsEBB1*, *PsEBB3*, and *PsBG6* were regulated by PsTOE3. Their expression levels during chilling-induced dormancy release were analyzed using qRT–PCR. *PsCYCD* was persistently induced after chilling treatment for 7–21 days, and its expression level increased ~2.32 times at 21 days. *PsBG6* was significantly upregulated by ~1.7 times until 14 days of chilling. *PsEBB1* expression significantly increased ~2.19 times after 7 days of chilling, followed by a continuous increase until 14 days and a continuous decrease until 28 days. The expression of *PsEBB3* was significantly increased by ~6.39 times after a chilling duration of 7 days, and then gradually declined until 28 days (Fig. 6A), which was consistent with our previous study [30]. Therefore, *PsCYCD*, *PsEBB1*, *PsEBB3*, and *PsBG6* responded to chilling treatment, suggesting that they might be involved in the same physiological process as *PsTOE3*.

Based on the genome sequence of tree peony [33], the promoter sequences of *PsEBB1*, *PsEBB3*, *PsCYCD*, and *PsBG6* were obtained using PCR (Supplementary Data File 2). Putative *cis*-elements were analyzed using the software PLACE; only one C-repeat (ACCGAC), an AP2 subfamily-specific binding consensus motif, was observed in the promoter of *PsEBB1*, from −613 to −608 bp (Fig. 6B). However, none was observed in the *PsEBB3* and *PsBG6* promoters. This suggested that PsTOE3 might act upstream of *PsEBB1* to regulate its expression. To verify this relationship, the full promoter of *PsEBB1* (F) was truncated into two fragments based on the C-repeat site: F1 (−2068 to −629) and F2 (−628 to −1). Three sequences were amplified and ligated with pHIS2.1 vector, and the encoding frame of *PsTOE3* was amplified and ligated into pGADT7 vector. The result of a yeast one-hybrid assay revealed that Y187 yeast cells cotransformed with pGADT7-*PsTOE3* and pHIS2.1-*PsEBB1F* could grow on SD/−His/−Leu/−Trp triple dropout medium with 80 mM 3-Amino-1,2,4-triazole (3-AT). The same result was obtained after cotransforming pGADT7-*PsTOE3* and pHIS2.1-*PsEBB1F2* (Fig. 6B). These results indicated that PsTOE3 protein could bind the promoter of *PsEBB1*, and the specific binding fragment contained the C-repeat. To further confirm that PsTOE3 could directly bind to the C-repeat, an electrophoretic mobility shift assay (EMSA) was performed using C-repeat as the probe. The recombinant vector of MBP-PsTOE3 was constructed, and the mutant C-repeat was used as the negative control. The result revealed that MBP-PsTOE3 could bind to the C-repeat of the *PsEBB1* promoter but could not bind to the mutant probe (Fig. 6C).

The regulatory effect of PsTOE3 on *PsEBB1* was further assessed using a dual luciferase (LUC) assay. Compared with the values obtained in the absence of PsTOE3, relative LUC activities indicated that the levels of firefly luciferase reporter (FLUC) were significantly increased when PsTOE3 was cotransformed into tobacco leaves with the *PsEBB1* promoter. The results indicated that PsTOE3 could promote the transcription of *PsEBB1*. Collectively, it was proved that PsTOE3 protein could bind to the *PsEBB1* promoter and activate its transcription (Fig. 6D and E).

## Discussion

Bud dormancy release is a prerequisite for forcing culture of tree peony in winter. Although recent studies on bud dormancy release in tree peony have provided understanding of the mechanism to some extent using transcriptome, microRNA, metabolomic, and epigenetic modification, etc. [27, 28, 31, 32], the regulation of bud dormancy is still poorly understood. MicroRNAs are non-coding RNAs and, along with their target genes, they are involved in many physiological processes in plants. miR172-AP2 mainly regulates flowering time and floral organ differentiation. In this study we observed that *PsmiR172b* targeted *PsTOE3*, and *PsmiR172b-PsTOE3* regulated bud dormancy release. PsTOE3 could directly activate the expression of *PsEBB1* at the transcriptional level. Meanwhile, heterogenous expressions of *PsmiR172b* and *PsTOE3* regulated seed germination and flowering in *Arabidopsis*. Our results demonstrated dual functions of the *PsmiR172b-PsTOE3* module in regulating bud dormancy release and flowering.

### *PsmiR172b* targeting *PsTOE3* regulates bud dormancy release in tree peony

It is well known that *miR172* and its target gene *AP2* regulate vegetative phase changes in perennial woody plants. *miR172* was reported to be involved in the cambial dormancy-active growth cycle in poplar [35]. However, it is still unclear whether *miR172* regulates bud endodormancy release. Based on our recent study on the differentially expressed microRNAs associated with bud dormancy [32], we analyzed the expression patterns of three peony *miR172* members, including *PsmiR172a*, *PsmiR172b*, and *PsmiR172d*, in this study. Among them, *PsmiR172b* was inhibited by prolonged chilling durations, which was basically consistent with the results with pre-*PsmiR172b*. This suggested that *PsmiR172b* might be involved in chilling-induced bud dormancy release (Fig. 1). When the plants were transferred to a greenhouse, the expression levels of *PsmiR172b* were opposite after chilling for 7 and 21 days. Bud dormancy was not released after chilling for 7 days, and the plants could not normally flower in the greenhouse. A total of 21 days of chilling was sufficient for dormancy release [28], and these plants could flower when they were transferred into the greenhouse. Furthermore, the function of *PsmiR172b* was studied; it was observed that the silencing of *PsmiR172b* promoted dormancy release and budbreak (Fig. 4), and heterogenous expressions of *PsmiR172b* regulated flowering in *Arabidopsis* (Supplementary Data File 3, Supplementary Data Fig. S3). This suggested that *PsmiR172b* negatively regulated bud dormancy release in tree peony, and *PsmiR172b* played different functions during dormancy and flowering.

The AP2 domain defines a large family of DNA-binding proteins and regulates diverse processes of development and metabolism of plants, including flowering, inflorescence, and meristem arrest [36, 37]. In this study we obtained one full-length *TOE3-*like cDNA sequence from the tree peony; its mRNA was dramatically upregulated at the early stage of dormancy release, and its protein levels exhibited a similar pattern [31]. We further confirmed that *PsTOE3* was a target of *PsmiR172b* according to their expression patterns and GUS activities after cotransforming tobacco leaves (Figs 1 and 3). Furthermore, *PsmiR172b* regulated *PsTOE3* at post-transcriptional level, and the cleavage site was identified using RLM 5′-RACE (Fig. 3). When *PsTOE3* was overexpressed in tree peony buds, budburst occurred early and buds grew faster (Fig. 5), indicating that *PsTOE3* accelerated bud dormancy release. These results were consistent with those obtained after the silencing of *PsmiR172b* in dormant peony buds, which further confirmed their target relationship and provided a direct proof that the *PsmiR172b-PsTOE3* module regulated endodormancy release. Furthermore, ectopic expression of *PsTOE3* in *Arabidopsis* proved that *PsTOE3* could promote seed dormancy release and germination (Supplementary Data File 3, Supplementary Data Fig. S3).

In contrast to the upregulation of *PsTOE3* during chilling, their transcript levels were dramatically reduced after moving into the greenhouse (Fig. 1). To study this in detail, *PsTOE3* was overexpressed in *Arabidopsis*, and it delayed flowering (Supplementary Data Fig. S3), which was consistent with the previous studies [6, 16, 35]. The results suggested that high expression of *PsTOE3* hindered plant flowering, and downregulation of *TOE3* was necessary before blooming. Taking these results together, we assumed that *PsTOE3* should be activated to assist peony bud dormancy release during chilling, and it should be repressed to ensure flowering after budbreak with regulation by *miR172b.*

### PsTOE3 accelerates bud dormancy release through the activation of *PsEBB1*

AP2 transcription factors are involved in many developmental processes in plants [37, 38]. In this study, *PsTOE3* could promote bud dormancy release, as indicated by genetic transformation evidence. The expressions of the genes associated with dormancy release, including *PsEBB1*, *PsEBB3*, *PsCYCD*, and *PsBG6*, in tree peony were increased in *PsTOE3-*OE buds and *PsmiR172b*-silenced buds. This suggested that *PsTOE3* might accelerate bud dormancy release by regulating the cell cycle or the reopening of a transport corridor. It is known that every subfamily of the AP2 family has a specific DNA binding preference, and the AP2/EREBP subfamily binding site contains a C-repeat (ACCGAC). We assessed whether PsTOE3 could regulate the transcription of *PsEBB1*, *PsEBB3*, *PsCYCD*, and *PsBG6*. To confirm this hypothesis, their promoter sequences were analyzed. Interestingly, we could not find the specific binding site of PsTOE3 in the promoters of *PsCYCD*, *PsEBB3*, and *PsBG6*, and one C-repeat was detected in the promoter of *PsEBB1*. In addition, *PsEBB1* was significantly induced by chilling duration. This suggested that PsTOE3 might directly regulate the transcription of *PsEBB1* during peony bud dormancy release. Yeast one-hybrid, EMSA, and dual luciferase assays confirmed that PsTOE3 could positively and directly regulate the expression of *PsEBB1* (Fig. 6)*.* In poplar, EBB1 and EBB3 are the positive regulators of budbreak. EBB3 is located downstream of EBB1 and can directly regulate *CYCD3.1*, revealing a possible regulatory module of temperature-mediated budbreak by activation of the cell cycle. Cell cycle arrest and renewal are important factors influencing the growth–dormancy–growth state transition [39]. For example, chilling treatment promotes cell proliferation and results in budbreak in balsam fir [40]. The cell-cycle regulators, including CYCDs and CDKs, regulated by environment and hormones are associated with the growth–dormancy cycle in poplar [20]. After chilling, proteins that accelerate cell proliferation and differentiation, including cell division cycle protein 48 and eukaryotic initiation factors IF4A-15 and 4A0, were upregulated in the shoot apical meristem of *Pinus sylvestris* L., suggesting that they might regulate bud dormancy [41]. After growth induction from paradormancy, the genes involved in the cell cycle are upregulated in leafy spurge root buds [42–44]. In grapes, for the transition from resting and endodormancy stages to a stage of active growth, an increase in cell division is needed [45]. In our previous study, genes associated with the cell cycle, including *CYCD*, *CYCA*, and *CYCB*, were upregulated at the end of endodormancy, which indicated that cell division is reinitiated and accelerated during dormancy release in tree peony [28]. Combined with the upregulation of *PsEBB1* and *PsCYCD* during chilling-induced dormancy release and in *PsTOE3*-OE buds, PsTOE3 could directly activate the expression of *PsEBB1*. Therefore, in this study, we present the idea that PsTOE3 activated the expression of *PsEBB1*; *PsCYCD* might be indirectly regulated by PsEBB1 and finally accelerated dormancy release and budbreak with the activation of cell cycle.

### Conclusions

**Figure 7:**
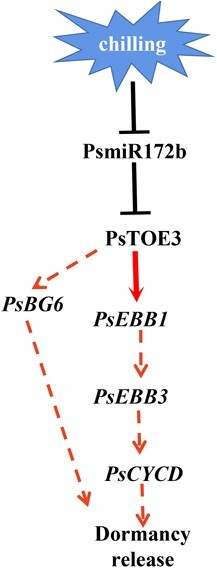
Hypothetical model of the roles of *PsmiR172b*-*PsTOE3* during chilling-induced bud dormancy release in tree peony. *PsmiR172b* responds to chilling treatment and is downregulated, and the expression of its target gene, *PsTOE3*, is induced during the same process. Both *PsmiR172b* silencing and *PsTOE3* overexpression lead to the upregulation of *PsEBB1*, *PsEBB3*, *PsCYCD*, and *PsBG6*; PsTOE3 can directly bind to the promoter of *PsEBB1* and activate its expression, indirectly accelerate cell proliferation and finally lead to bud dormancy release and budbreak. Red arrows represent positive regulation, black bars represent negative regulation. Dotted red arrows indicate indirectly positive regulation.

Among the three miR172 family members, the expression of *PsmiR172b* was downregulated during chilling-induced dormancy release. *PsTOE3* was its target, as confirmed by GUS activities after cotransformation and RLM 5′-RACE. The *TRV2-STTMPsmiR172b* and *PsTOE3-OE* buds grew faster than the control, and the genes associated with dormancy release in tree peony, including *PsEBB1*, *PsEBB3*, *PsCYCD*, and *PsBG6*, were upregulated. PsTOE3 could directly bind to the promoter of *PsEBB1* and activate its expression as revealed by yeast one-hybrid, EMSA, and dual luciferase assays. Collectively, prolonged chilling inhibited the expression of *PsmiR172b* and increased the transcript levels of target gene *PsTOE3*. PsTOE3 could directly activate *PsEBB1*, which might finally accelerate cell proliferation and lead to bud dormancy release and budbreak (Fig. 7). After bud dormancy release, *PsTOE3* needed be downregulated to ensure the subsequent flowering. Our results present a novel function of *PsmiR172-PsTOE3*, which is beneficial to understanding of the mechanism of dormancy release regulation.

## Materials and methods

### Plant material and treatment

For this study, 4-year-old tree peonies (*P. suffruticosa* ‘Luhehong’) were obtained from Qingdao Agricultural University (Shandong, China). Based on our recent study [28], 21 days of chilling was adequate for dormancy release of ‘Luhehong’, and the physiological status of buds after chilling for 28 days was ecodormancy. The tree peony plants were treated at 4°C (24 hours of darkness) for various time periods (0, 7, 14, 21, and 28 days) based on our previous methods [28, 46]. Further, their buds were sampled and immediately put into liquid nitrogen, and stored at −80°C until use. After chilling treatments for 7 and 21 days, the plants were transferred to a greenhouse (22°C, 16 hours light/8 hours darkness), and the buds were collected after 0, 1, 3, and 7 days and cryopreserved in liquid nitrogen. Each treatment was performed in triplicate, and each replicate had three plants.

Other plants without sampling were moved to the greenhouse to evaluate their dormancy status as previously described [30].

### DNA and total RNA isolation

Genomic DNA was extracted from tree peony buds using the cetyltrimethylammonium bromide (CTAB) extraction method as previously described [46]. Total RNA was isolated using RNAiso Plus (TaKaRa, Dalian, China) according to the manufacturer’s instruction and digested with RNase-free DNase Ι (TaKaRa, Dalian, China) to remove the residual genomic DNA. RNA purity and concentration were checked using a SmartSpec™ Plus Spectrophotometer (Bio-Rad, USA).

### Rapid amplification of cDNA ends

RACE amplification was performed to obtain the full-length cDNA sequence of *PsTOE3*. The primers PsTOE3GSP5′ and PsTOE3GSP3′ were designed for RACE amplification based on the partial *AP2* EST sequence from RNA-seq sequencing (Supplementary Data Table S1) [46]. cDNA synthesis and RACE PCR were performed using the SMART™ RACE cDNA amplification kit (ClonTech, USA) according to the manufacturer’s instructions. The objective fragments were collected using DNA and a gel extraction kit (Tiangen, Beijing, China), and then ligated into pMD18-T vector (TaKaRa, Dalian, China), and propagated in *Escherichia coli* DH5α. The positive colonies were sequenced by Sangon Co., Ltd. (Shanghai, China).

### Bioinformatic analysis

Sequence assembly was performed using DNAMAN 9.0. The nucleotide sequence and the deduced amino acid sequence were compared using the BLAST program, and the ORF was identified using ORF Finder at NCBI (http://www.ncbi.nlm.nih.gov/). The subcellular localization was predicted with WoLF PSORT (https://www.genscript.com/tools/wolf-psort). The multiple alignment was performed using ClustalW with default parameters. The phylogenetic tree was constructed based on the neighbor-joining (NJ) model with 1000 bootstrap replications using MEGA 11.0.

Based on the genomic sequence of tree peony [33], the precursor of *PsmiR172s* was obtained, and the secondary structure was predicted using Tbtools V1.098696. The data for known miR172 family members in other plants were downloaded from miRBase (https://www.mirbase.org/).

### Real-time quantitative RT–PCR

To detect the expression patterns of *PsmiR172s* and pre-*PsmiR172s*, sRNAs were isolated from tree peony buds using RNAiso for Small RNA (TaKaRa, Dalian, China). The first-strand cDNA was amplified using the SYBR^®^ PrimeScript miRNA RT-PCR Kit (TaKaRa, Dalian, China). qRT–PCR was performed according to our recent method with *PsU6* as the internal reference [32]. The primers used are listed in Supplementary Data Table S1.

The first-strand cDNA was synthesized from 2 μg total RNA using the PrimeScript™ RT Reagent Kit (TaKaRa, Dalian, China). The primer pairs of *PsTOE3* and the other genes associated with dormancy release, including *PsCYCD*, *PsEBB1*, *PsEBB3*, *PsBG6*, and *PsActin* (as the internal reference), are listed in Supplementary Data Table S1*.* PCR was performed on a QuantStudio™ 5 Real-Time PCR instrument (Thermo Fisher, USA) using the SYBR^®^ Premix Ex Taq™ II Kit (TaKaRa, Dalian, China). The PCR program was as follows: 95°C for 2 minutes and 40 cycles of 95°C for 5 seconds, 55°C for 30 seconds, and 72°C for 30 seconds. The relative expression levels of each gene were assessed according to the 2^−△△Ct^ method [47]. Significance was tested using SPSS 13.0 for Windows (SPSS, USA).

### Anti-PsTOE3 antibody preparation and immunoblot analysis

The putative protein of PsTOE3 was BLASTed with the *Arabidopsis* AtAP2 protein (Supplementary Data File 4). The specific peptide fragments, including P1 (11–30 amino acids), P2 (48–81 amino acids), and P3 (344–363 amino acids), were synthesized, and their antibodies were obtained by inoculating mice (ABclonal, Wuhan). Total proteins were extracted from the buds after different chilling treatments [48], and 100 μg of each protein sample was denatured by boiling, separated on a 10% SDS–PAGE gel, and transferred to a polyvinylidene fluoride (PVDF) membrane. After blocking the membrane for 2 hours, the levels of PsTOE3 and PsActin were determined.

### Subcellular localization of PsTOE3

The *PsTOE3* ORF without stop codon was amplified with the primers PsTOE3-GFPF and PsTOE3-GFPR (Supplementary Data Table S1). The GFP-PsTOE3 fusion expression vector was constructed and used to transform *A. tumefaciens* EHA105. *Agrobacterium* was cultured in LB liquid medium (with kanamycin and rifampicin) until an OD_600_ of 0.6–0.8 was obtained. Further, the cells were centrifuged at 5000 rpm for 10 minutes. The cell pellet was diluted to obtain an OD_600_ of 0.6–0.8 with a solution of 10 mM MES, 200 mM acetylsyringone, and 10 mM MgCl_2_ (pH 5.6), and injected into tobacco leaves. The infected plants were cultured in darkness for 1 day, and then in normal conditions (25°C, 16 hours light/8 hours darkness) for 1 day. DAPI staining was performed to stain the nucleus, and the fluorescence was observed using a laser confocal microscope.

### Transient transformation in *Nicotiana benthamiana* leaves and GUS activity measurement

To further study the target relationship between *PsmiR172b* and *PsTOE3*, 230-bp *PsmiR172b* precursor was amplified (Supplementary Data File 1), and the fusion expression vector *PsmiR172b:GUS* was constructed with pSuper1300 vector. The *PsmiR172b* targeting sites in *PsTOE3* were replaced with the synonymous mutation bases (*mPsTOE3*) as the negative control (Fig. 2), and the 35S::*PsTOE3:GUS* and 35S::*mPsTOE3:GUS* fusion expression vectors were constructed. The primers used are listed in Supplementary Data Table S1. These three vectors were used to transform into *Agrobacterium* GV3101, which was used to infect *N. benthamiana* leaves. After 3–4 days, GUS histochemical staining was performed using the method described by Jefferson *et al*. [49]. GUS activity was evaluated as picomoles of 4-methylumbelliferone produced per minute per milligram of protein. Protein concentration was determined using a protein assay kit (Bio-Rad, Hercules, CA, USA) with bovine serum albumin as the standard.

### RNA ligase-mediated 5′-RACE

RLM 5′-RACE was performed to determine the cleavage site of *PsmiR172b* in the *PsTOE3* cDNA sequence using the FirstChoice^®^ RLM-RACE Kit (Ambion, USA). Specifically, total RNA (obtained from buds after chilling for 14 days) was ligated with the 5′-adapter, and the ligated RNA was used to synthesize the cDNA. Based on the complementary region of *PsmiR172b* at *PsTOE3*, RLM 5′-RACE primers were designed (Supplementary Data Table S1). The 5′-RACE cDNA was used as the PCR template according to the manufacturer’s instructions. The PCR products were purified and ligated into pMD18-T vector (TaKaRa, Dalian, China), and 20 positive monoclonals were randomly selected and sequenced.

### Yeast one-hybrid assay

The promoter sequences of *PsEBB1*, *PsEBB3*, *PsCYCD*, and *PsBG6* were obtained via PCR amplification according to the genomic sequence of tree peony [33]. Their promoter sequences were analyzed using the online software PLACE (http://www.dna.affrc.go.jp/PLACE).

The *PsTOE3* ORF was amplified and ligated to pGADT7 vector with restriction enzyme sites of *EcoR*I and *Nde*I. The *PsEBB1* promoter was designated as F, and was truncated into two fragments based on the C-repeat site: F1 (−2068 to −629) and F2 (−628 to −1) (Fig. 6) (Supplementary Data File 2). Three fragments were amplified and ligated into pHIS2.1 vector with restriction sites of *EcoR*I and *Mlu*I. The recombinant plasmids (pGADT7 + pHIS2.1-*proPsEBB1F*, *proPsEBB1*F1, and *proPsEBB1*F2) were transferred into yeast Y187. After culturing in darkness at 29°C for 3–5 days, a single colony was diluted 10 and 100 times, and the optimum 3-AT concentration was screened on SD/−His/−Leu/−Trp medium. Finally, the recombinant plasmids (pGADT7-*PsTOE3* + pHIS2.1-*proPsEBB1F*, *proPsEBB1F1*, and *proPsEBB1F2*) were used for cotransformation into Y187, and the transformants were observed on SD/−His/−Leu/−Trp medium at the corresponding 3-AT concentration.

### Electrophoretic mobility shift assays

Fusion expression vectors, including MBP-PsTOE3, were constructed and used to transform *E. coli* BL21. The positive colonies were cultured at 37°C and 200 rpm until an OD_600_ of 0.6–0.8 was obtained. Further, 0.1 mM IPTG (isopropyl β-d-thiogalactoside, isopropyl-β-d-thiogalactopyranoside) was added to induce the expression of fusion proteins; after this, the cells were cultured at 18°C and 150 rpm for 15 hours. The culture was centrifuged at 4000 rpm at 4°C for 15 minutes, and the cell pellet was suspended in binding buffer (20 mM Tris–HCl, 0.2 M NaCl, 1 mM EDTA, and 10 mM β-mercaptoethanol). The fusion proteins were purified from the inclusion bodies on Ni^2+^-NTA agarose resin (Qiagen) according to the manufacturer’s protocol.

The C-repeat (ACCGAC) was synthesized with four replications. The double-stranded probes were prepared by annealing and marked with the DIG Gel Shift Kit (Roche) with the final concentration of 0.155 μM. Poly(dI-dC) was used as a non-specific DNA competitor. EMSA was performed using a DIG Gel Shift Kit (Roche) according to the manufacturer’s instructions. As the negative control, the mutation C-repeat (AGCGAC) was synthesized with four replications. The primers used for EMSA are listed in Supplementary Data Table S1.

### Dual luciferase assay

The ORF of *PsTOE3* was obtained and was inserted into binary vector pBI121. Meanwhile, the promoter of *PsEBB1* was cloned into pGreenII0800-LUC vector. The recombinant plasmids were introduced into *Agrobacterium* GV3101 and cultured at 28°C in LB liquid medium with kanamycin and rifampicin. The bacterial cells were injected into *N. benthamiana* leaves. Four days after injection, the dual luciferase assay was performed for enzyme activity determination in leaves. FLUC and REN (*Renilla* luciferase) were assayed using a Dual-Luciferase Reporter Assay Kit (Vazyme), and their activities were measured using an ultrasensitive multifunctional microplate reader (Cytation 5, BioTek) and a plant *in vitro* fluorescence detector (Newton7.0, Vilber). The activity of REN was considered as the reference to normalize the activity of FLUC. Each treatment involved five biological replicates and three technical replicates.

### Transformation of tree peony buds

To further elucidate the function of *PsmiR172b*, *PsmiR172b* was knocked down using a TRV-mediated short tandem target mimic (STTM) approach [50, 51]. The gene-specific fragment of *STTM172b* (STTM of *PsmiR172b*, 96 bp) was used to construct the plasmid TRV2-*STTM172b*. Related information on TRV2-*STTM172b* is listed in Supplementary Data Fig. S4. TRV1, TRV2, and TRV2-*STTM172b* were used to transform *A. tumefaciens* EHA105, which was cultured in LB medium (with 40 mg l^−1^ kanamycin, 20 mg l^−1^ gentamicin, 10 mM MES, and 20 μM acetosyringone) at 28°C for 48 hours. *Agrobacterium* cultures were centrifuged at 4000 rpm for 20 minutes and diluted using a buffer containing 10 mM MgCl_2_, 10 mM MES, and 200 μM acetosyringone until a final OD_600_ of 1.0–1.2 was obtained. TRV2 and TRV2-PsmiR172b were separately mixed with TRV1 according to the volume ratio of 1:1 and allowed to stand for 4–6 hours in darkness. The buds were sterilized after 7 days of chilling and submerged in the infiltration buffer for 3–4 minutes in a vacuum dryer at 0.3 MPa using a vacuum pump and slowly deflated for 30–40 minutes. These buds were transferred into 1/2 Murashige and Skoog (MS) medium with 200 μM acetosyringone under aseptic conditions. After dark treatment for 4 days (8°C for 3 days followed by 22°C for 1 day), the buds were transferred to MS medium (with 200 mg l^−1^ ticarcillin and 0.5 M MES) and cultured at 22°C (16 hours light/8 hours darkness). After infection for 10 days, qPCR was performed to detect the silencing efficiency of *TRV2-PsmiR172b* buds, and the morphology changes including the relative growth of bud width and height were observed and measured every day. In total, 60 buds were used per transformation, with 30 buds for expression detection and 30 for morphological observations.

The *PsTOE3* ORF was amplified, digested with *Sma*Ι and *Sac*Ι, and inserted into the binary vector pBI121, in which the CaMV 35S promoter drove the transcription of *PsTOE3*. The primers use are listed in Supplementary Data Table S1. Further, the pBI121-*PsTOE3* vector was transformed into *A. tumefaciens* EHA105, and after 7 days of chilling the sterilized tree peony buds were infected with pBI121-*PsTOE3* according to the above method. PCR, qPCR, and western blot were performed to detect positive transgenic *PsTOE3* buds, and the morphology changes were observed and measured according to the above method.

## Acknowledgements

This work was supported by grants from National Natural Science Foundation of China (31872145 and 31972452) and the National Key R&D Program of China (2018YFD1000403). The funding bodies had no role in the design of the study, the collection, analysis, and interpretation of data, or in writing the manuscript. The authors would like to thank MJEditor (www.mjeditor.com) for providing English editing services during the preparation of this manuscript.

## Author contributions

G.S. and Z.Y. conceived and designed the experimental plan. W.Y., G.L., and N.D conducted the experiments. Z.Y., Y.Y. and Z.X. analyzed the data. Z.Y., L.C., and G.S. prepared and revised the manuscript. All authors have reviewed and approved the final manuscript.

## Data availability

The sequence data that support the findings of this study are available in the NCBI and TAIR databases with the following accession numbers: PsTOE3 (KJ777535), PsEBB1 (OP095871), PsEBB3 (OP095872), PsCYCD (OP095873), PsBG6 (OP095874), AtCYCD1;1 (AT1G70210.1), AtCYCD2;1 (AT2G22490.2), AtCYCD3;1 (AT4G34160.1), AtCYCD3;3 (AT3G50070.1), AtCYCD4;1 (AT5G65420.3), AtCYCD7;1 (AT5G02110.1), AtSOC1 (AT2G45660.1), AtFT (AT1G65480.2), AtLFY (AT5G61850.2).

## Conflict of interest

The authors declare no competing interests.

## Supplementary Data

Supplementary data is available at *Horticulture Research* online.

## Supplementary Material

Web_Material_uhad033
