# Supplementary material for: Dual functions of PsmiR172b-PsTOE3 module in dormancy release and flowering in tree peony (Paeonia suffruticosa)
Source: Hortic Res. 2023 Feb 21;10(4):uhad033. doi: 10.1093/hr/uhad033 (PMC10120838; doi:10.1093/hr/uhad033)
Supplement: Web_Material_uhad033 [file web_material_uhad033.zip › clean supplemental file 3.docx]

**Supplemental file 3**

**Methods**

**Transformation of Arabidopsis**

pBI121*-PsTOE3*, pBI121-*PsmiR172b* vectors were constructed and transformed into wild type Columbia Arabidopsis (Col-0) using the floral dip method. The T_3_ generation *35S::PsTOE3* homozygotes were hybridized with that of *35S::PsmiR172b*, and obtain the F_3_ homozygous hybrid lines of *35S::PsmiR172b+35S::PsTOE3*. PCR and qPCR were carried out to confirm the transgenic plants (Table S1). These T_3_ transgenic seeds were treated with 4℃ (in darkness) for 2 d, and the germination rates were measured. Each treatment repeated three times, and each replicate had at least 50 seeds. The expression of *AtCYCD3;1* was detected by qRT-PCR, *AtActin* was used as internal reference (Table S1).

To investigate flowering time, the numbers of rosette leaves were counted when the T_3_ transgenic and wild type Arabidopsis plants grew to a 1-cm-long inflorescence at long-day (LD) conditions (20-22℃, 16 h light/8 h dark cycle). The expressions of the positive flowering regulation genes including *AtFTF*, *AtSOC1* and *AtLFY* were detected with the specific primers, *AtActin* was used as internal reference (Table S1).

The data were analyzed using SPSS 13.0 for Windows (SPSS, USA).

**Results**

**Heterologous expressions of *PsmiR172b* and *PsTOE3* in Arabidopsis regulates seed germination and flowering**

The ORF of *PsTOE3*, and a 230 bp precursor sequence of *PsmiR172b* were respectively amplified and inserted into pSuper1300 overexpression vector, then were transformed into wild type *Arabidopsis thaliana* by flower dipping method. T_3_ homozygotes (*35S::PsTOE3*, *35S::PsmiR172b*) were obtained. qRT-PCR showed that the transcripts of *PsTOE3* were inhibited in *35S::PsmiR172b+35S::PsTOE3* plants (Fig. S3A)*.*

The seed germination rate of T_3_ *35S::PsmiR172b*, *35S::PsTOE3* and F_3_ homozygous hybrid lines of *35S::PsmiR172b+35S::PsTOE3* were calculated, and found that overexpression *PsTOE3* significantly promoted seed germination, while *PsmiR172b* significantly inhibited seed germination. Additionally, the seed germination rate of *35S::PsmiR172b+35S::PsTOE3* lines was also inhibited, which was lower than that of *35S::PsmiR172b* lines (Fig. S3B, C). According to our results, after silencing of *PsmiR172b* and overexpression of *PsTOE3* promoted the expression of *PsCYCD* in peony, thus the putative PsCYCD protein sequence was aligned and clustered with AtCYCD proteins including AtCYCD1;1, AtCYCD2;1, AtCYCD3;3, AtCYCD4;1 and AtCYCD7;1, and we found that PsCYCD was close to AtCYCD3;1 with 55.4% identity. Therefore, the expression level of *AtCYCD3;1* was analyzed by qRT-PCR, and *AtCYCD3;1* in *35S::PsTOE3* T_3_ transgenic seeds were higher than that in *35S::PsmiR172b* and *35S::PsmiR172b+35S::PsTOE3*, which were consistent with the germination rates (Fig. S3D).

In addition, the flowering morphology indexes including rosette leaf number, flowering time were also calculated. The results showed that the rosette leaves number of *35S::PsmiR172b* and *35S::PsmiR172b+35S::PsTOE3* plants were 3-4 fewer than control, while those of *35S::PsTOE3* plants were 1-2 more than control (Fig. S3E, F). The flowering time of *35S::PsmiR172b*, *35S::PsTOE3* and *35S::PsmiR172b+35S::PsTOE3* transgenic plants were 20.87 d, 24.66 d and 20.93 d, respectively. The transgenic plants of *35S::PsmiR172b* and *35S::PsmiR172b+35S::PsTOE3* showed earlier flowering with no significant differences. Therefore, the results showed that *PsmiR172b* played an importantly conservative role as flowering activator by inhibiting the expression of *PsTOE3*. The known positive regulators of flowering including *AtFT*, *AtLFY*, and *AtSOC1* were dramatically induced in *35S::PsmiR172b* and *35S::PsmiR172b+35S::PsTOE3* plants, but inhibited in *35S::PsTOE3* transgenic plants (Fig. S3G).

Altogether, we assumed that *PsmiR172b* and *PsTOE3* were multifunctional genes, which involved in seed dormancy, bud dormancy release, flowering and other potential unknown function.
