# Supplementary material for: Dual functions of PsmiR172b-PsTOE3 module in dormancy release and flowering in tree peony (Paeonia suffruticosa)
Source: Hortic Res. 2023 Feb 21;10(4):uhad033. doi: 10.1093/hr/uhad033 (PMC10120838; doi:10.1093/hr/uhad033)
Supplement: Web_Material_uhad033 [file web_material_uhad033.zip › Supplemental file 1 Premir172a, b d.docx]

**Supplemental file 1**

**Pre-PsmiR172a**

UAACCACUAGUAAAAGGCCACAUCAUCAUAUGCUGGUGUUACUGAGUAUGAAUGGGUACUUUUGGCAUUAAUGGAGGACGGUUGUUGUUUGCUGGU**GUAGCACCAUCAAGAUUCACA**UGAGAAUUUUUGCCAGCUCCGGUGGAAAUGACCCUAGAUCUACCAAUGUCUUGAACGUG**AGAAUCUUGAUGAUGCUGCAU**UGGCAAUAAACAACUAAACAAAGCUCAACCGUGCCACUUUAAAGGUAUAUAUAUAUAUAUAUAUAUAUAUAUAUAUAUAUAUAUAUAUAUAUAUAUAUAUUAUAUAUAUUAUGUCUAGUUAAUUCUAUGUGGGGACUGGGGUUGUAAUUUUUUUGUAUGCAUA

**Pre-PsmiR172b**

CAUCCACAAACUUUACGUUUUAACCACUAGUAAAAGGCCACAUCAUCAUAUGCUGGUGUUACUGAGUAUGAAUGGGUACUUUUGGCAUUAAUGGAGGACGGUUGUUGUUUGCUGG**UGUAGCACCAUCAAGAUUCA**CAUGAGAAUUUUUGCCAGCUCCGGUGGAAAUGACCCUAGAUCUACCAAUGUCUUGAACGUG**AGAAUCUUGAUGAUGCUGCAU**UGGCAAUAAACAA

**Pre-PsmiR172d**

CCGUUUUAGUUUCUGUACGUGUAGCAGUAGGAAGUGUUGGCCACAGAAGGAAGGAAGACAUUCUGAGAAGUACUGUUCUGCGGGUAGAGAAGCGGAAGGGUAGCCGGAGAUGA**AGCACAAGGGUUAGAA**GCAG**AGAAUCCUGAUGAUGCUGCA**CUGGAGAACAUUGGCAUUGGCGUGACGCCGCUAUUGCGAUACAUUUGCCAUGCAUAGUUUGAGAAUCUUGGCAAAGAAGUGGCUUCAACUCUGUUCUCAGCAGCUCGAUCCUGCUUAAUUAAAAAAAUUAAUUAAUCAAAAUUAAGGAAAUGGGACAGAAAGUUCACAAAUAAAUAUUGUAUCGGGAGUAGCAUCAUGUUCAAGCAA
