# Supplementary material for: Dual functions of PsmiR172b-PsTOE3 module in dormancy release and flowering in tree peony (Paeonia suffruticosa)
Source: Hortic Res. 2023 Feb 21;10(4):uhad033. doi: 10.1093/hr/uhad033 (PMC10120838; doi:10.1093/hr/uhad033)
Supplement: Web_Material_uhad033 [file web_material_uhad033.zip › Supplemental file 2 promoter of PsCYCD.docx]

**Supplemental file 2**

**A.The promoter of *PsEBB1***

-2068 GGGATGTAAG CCAAATGCTC TCTATAAATA AGAGGAGCTC TAGGAGTGTA

**F1**

-2018 AAACCCTAAG AGAAATTCGG CCATATGTGG GTGTCACTTT CTCTCTCTTA

-1968 AATCCTAGAC CCCCTAAACT TTCTAACAAT ATTTTATATT TTTTACATAT

-1918 TATTAAACAA TGAGGAACTT AGGGCGGGCT TGAAGATAGA GCAGACTTAC

-1868 CGTTCGAGTT TGATTTTTAG GGTCAGACAA TGGCCGCACC CTTAGAACTT

-1818 GAAGGCCGCG TTTGTTTCGT GGTTTCAACC AAATAAGACC GGGACTAATA

-1768 TTAGCCGAAC ACTGTTCATC GGCTAATCCT TCCTCTGTTG TCTGTTTGTT

-1718 TTGAGTTTTT GGAAAATCAA TCCCAGCCTT AACCCTTTAA CACCGGGCTT

-1668 AGCTTCCTTT TTTAACACCA CCAAAATCGG TGTGTTTGCA AAACAACACC

-1618 GGGCTTAAGT TTTTTCTTTC TTTTCTCCTC TCCTCTGCAA CATCATCCCT

-1568 CTCCTCTGCA ACATCATCAC GCCGGAGCAG CATATCTTTC CGTTTCTCAT

-1518 CTCCTCTGCA AATCATCATT CTCCTCTGCA ACATCATCAT GCCGGAGCAG

-1468 CAGCATCTTG CCGAAATCTG CATCACATCA TCGGTTTGAT ATCATCGGTT

-1418 TGATCTTAGT TCCTTGAAAA CACCAAATTT TCTTGCCTTG AGATAATGAT

-1368 CGGTACTTTG TAATTTGTTT TGAGATTTGT TGGTGATGAA TATACACACG

-1318 AGATCGGTAC TTTGTAATTT TTCTTGTGAT CTGTTTTTGA GATCAGTACT

-1268 TTTTGATTAT TTTTTTTTGT GATCTGTTGG TGATGAAATA CACGAGCATG

-1218 ACTTTTATAT ACACATGCGC TCATGGCTTT TATCTGTTGG TGATGGATAT

-1168 ACAGGGGATG ACCTTAGTCA GATCTGTTGG TGATGAATGT TTTGTTGATA

-1118 TATAATATAT ATATATATAT ATATATATAT AGATATATAT ATATATATAT

-1068 ATATATATAT ATATAGATAT ATATATATAT ATATATAGTT ATTCGAGCAT

-1018 GATTTTTTGC CCATTTTATA CCTCTGTTTT TATATATATA TATATATATA

-968 TATATATATA TATATGGTAT TTTGGTAGCA CTAATCCCAT GCTTATTTAC

-918 TCTTTCAAAT AAACACTATG CAAAAAAATA GTCTCAGGCT TAGCCACCAA

-868 CAAAAAAAGA ATCCCATGCT TAGCCATCAA AATTTTGGCT AATCCCATTG

-818 GCTAAACTCG GGCTTATTTT TTTTTACTAC TCCAAACAAA CGCTAACGAA

-768 AGGCTTACGG GCTGGGCCAA TCATCATTTT TTTTTTTTTT TTACTGGCAT

-718 TAGTCGTGCG AAATATGATG GGTCGGCAGG CGATTTGAAA TTTCTACCCA

-668 TCCCACACTC ACATGATGAG CATTTGCTAT CACGTGACTG CTTTGGACTT

**F1 F2**

-618 TTGCA**ACCGA C**CAACAGACT TAGCAAAATA CTCCCTCGTA TGTGAGTAAT

**C-repeat**

-568 CAATATACAT CCTTCATAGA GAGCGAGTCC CCTTTTCTTG CTAGGCGAGC

-518 GAGTATAATT TACAAAAGAT GTTGTTACCA GGTTACAAAT AAAGTCTGCC

-468 CATCATATTT AACATAAAAA TCTAACGGCG AGTCGATGAA AAAAAAAAAA

-418 TACAAAAAAT CTTCGGTACA CGCAATAACA CCTCTGAATC TCTGATAAAT

-368 TTTCTTTACT CTCTCTCTCT CATGCCTCAT GGCCACCTCT CTCTGCAACC

-318 ACTGAGCATA AATGAAAGGA GGAACGAAAC CCAATGCTCC TTCTCTCGGT

-268 GCTAGCAGCT AGAGATTGAA ATGAGATTCG AATTATTTAT TTATTTTTAT

-218 TTTTATTTTT ACATGAAACA TACATCTGTA TCTAACATTT TTACAGACTT

-168 ACAAATCAGG GTTTTATGAT GACAAAGTTT TCTCTTTGTA CGTTTTTTTC

**CAAT-box**

-118 TATAATTACT TCAAAACCCA AAAACTCACC AGCTTTCAAG TTTGCTTGAG

**TATA-box**

-68 CTCAAAACCC AAAAACTAGG TTAAAAGACA ACACACATAG AGAAAGAGTG

-18 AGAGAGGAGA CAGAGAAT**AT G**

**F2**

**B.The promoter of *PsEBB3***

-2048 GCCTAGGTTA TGTGGCGAAC TTAGTATCCC TAGTCTTTAT TCTCTTGAAT TTGATTACTT

-1988 TAAAATTACT TGTTTTAGTT TAACTTATAA TTTTCTCAGA TTTTAAACGT CTTTTAAACA

-1928 AATCTCTTGA CCTTGTTCGC TTGAGTAACT TTTAGCATAC ATTTCATTTC TCAATGTGGA

-1868 TTCAACCTTG TTTATTTTAA TGCATTACTA CCCCAATCTT GTGTTAGTTG GCAAGTAAAA

-1808 GTGAATCTTT TCAATCTTTT ACTTTTTACA TATATTTATA TACATTAGGA GGAGACTCGT

-1748 CAAGTTTTGT GCAAGATAGA TAGTGGGATA GGTACAATTA AAAAAAAGAA GCCTTCTTCA

-1688 TCATTGTTTA ATTGGAGAGT AGATAAACTG TTGTGCTCTC TTGGAGTGCG ATTCGATGTT

-1628 GTTCAACAAA AGGATTTGTG TGATTCGTAC CTAGAGAACT TGGTGTGTTC AATGTAAGGA

-1568 GTTGATGTGA TTTCAATTTG GAATATGAGT TAATCGTATC GACAGTTTGC TAGTTATTCA

-1508 ATTGATGCGT TGTAGTGCTA GGTGCGAGCC GCGGTTCAAC TAGTTAGACT TAAACTTTAG

-1448 TTGCTATAAT TAAGTGATTT AATTAATCTA AAGATTATAC CAATTTATAG TCTATTATTA

-1388 AATGACTTAA TTTTAGTGGA GACCAAACGC ACCGTGGAGA TGTGTACCGT GGACATAGGT

-1328 TAAGGAGACC GAACCACGTA ATATCTTGTG TTGATTATTT GATTTCTATA TTTGGTGATT

-1268 TATGTATGTT TTCATAATCA CTGCACGAAA TTAGATTGAT TAAAGGCCAG ACTTAATTAT

-1208 TTGAATTAAG CAATCATTAG CACAATCAAA AATTATTATT TTTCATTACA AGCGATCAAT

-1148 TATAGTAGAA CATTTGGCGT GAATGATTTA AATCATTTAT TTTTAAATGG TATTGTCAAT

-1088 CCTAAAAAAA CCAACATATT TACCCTAGGA CCAGCCCTGG ATATGAGTCG AAGAAGAAAC

-1028 AAAAACAAAA ACTTGGAAAC GTAGGAGATT CTATTAACAT TTTCTTTTAG AAAGTAGAGG

-968 GTTCTTGTTA TCCAAAAATG AAAACTAAAA AAATGAAGCC CAAAGCCAAG TCTAAAAGTA

-908 GTGTTTTATG ACGTTATCAC ATACTCAAAA TAGTAAATCC ATCATAACAC AAATCACATA

-848 CACAATTCAT CATAAACACA AAATATATTC CTATTTTCAC TTAGAAATTT CATAAAATAG

-788 GTGAAATACA TATTTCAACA CATCAACATA TAATATAATT AAAGAAAGGG TACAAAATAG

-728 CCACCTACGC GTTTCGTCAA ACAAGTGAAG TGCACGATTA ATGTCTTGCA CAATTGACAA

-668 AGGTTATATA CAATTATACA TATCTTTTTC CACTAGGATG ACGTCGAAAA TTGTTAATTT

-608 ACGGTGAATT GAAACCAAAA GAACATGGAG TTTTAATGTG CTTTCTAATT GTGTTTGTGA

-548 TTTTTTGCTT CTTTAAAAAA TATGTTAGTT CATCAAATAG AAGGTATACA TGTGTAATTG

-488 ACTCATAATG ATGGGATGTA CCAAGTAAAT AACTCAAAGT TTGAACAAAT GTCTAATGCC

-428 ACATGTCCAT AATTTAGGCC TATTGCATGC CAATTGCATA CATTGATGGG TAAACTTATC

-368 AGTTAGCACT GCTTTTTGCA CACTAAAAAA ATAAGAAGAT AAATCTTACG CTCTCCTCTC

-308 ACCGATGAAA ACCCTTATTT GAGTATTTTC CTTACCTGAT GGTTTTTCTT CATTTCCTCC

-248 CAGCACGTCT ATCGTTACCT ATATGATCTG ATCCCAACAA ACAAACCAAC CTCCAAACTA

-188 AAGAAATAAA CAAGTCCGGG CTCTAGTTAG TTGATAAATA TTAGAGAGAG AGTTTTTTTA

-128 AGCTCATAAC GGTGGATCGA AGGCATGGAA AGCGGCCACT ACCTTCGGAA TCATCGGAAG

-68 AGAAAGAGAA AAAATCCTTC CCTCCTCAT**C AATAT**GTAAC TTCTTCTTCT TGGGCCCAAA

**TATA-box(underline)**

**CAAT-box(double underline)**

-8 ACGACGCC**AT G**

**C.The promoter of *PsCYCD***

-2318 AGCCGTGGAA AATCAATTGG TTTCCCCCTG ATAACTGCGT TTGGTACGCG

-2268 GTGAAGCTTC TCCGGCGGCA ACAACAGTCC CAGATAAACA GGTTTTATCC

-2218 GGGCAAACTT ATCCGGTCCT TTTGGGACGG ATAGAGTTTT TATCCCTGGA

-2168 TAAACTCTAT TTCCCTTATG CCCCCTTTTT ATCTCCTAAA TAAAAATTAT

-2118 AAGGGGCTAT TTAGTAAAAC TTATAATTAT CCCTACATTA TTTTTTATTC

-2068 TACCAAACGG TATTTATCCC TGACATAATT TATACATTTT TGGCTTATCC

-2018 CGAGGGCAAA TGGAAGATGA GCTTGGCCAT CGTGGTTTTT GCCTCTCCAA

-1968 ACACCTCCCA AATTAACGCT GGTGTTCAAA CTTTTACTCA GAACTTAAGA

-1918 GTGCTAGTGC CTAAAATACT ACCACAATGG GAACTACTTG AGTTCATAGC

-1868 TGTAAATTTC ATTTGAAACA TAAGGAATCG CCACAAAGGA AGAGTGGATG

-1818 CTGGTTTGAA TCTTAAGACT GGTACCTGAA GATGGCGCGG CCAAAAGCGA

-1768 TCTTAAATTT TAACGTTGGT AGGATTAGAT CAGCTTGTTG CGCGTCGATT

-1718 AGAAGACTGC GATGATTTCT GTCGATAAAG ACAGCAGCAA GATGAGCTTT

-1668 TCGATATGAA TCACAATCCT CAATTTTCAA TTTTTTTTGG TGTCACTTAC

-1618 ATGCATCCCA CACTTTGATT TCTTCGTCCC ACACTTTCAT TTTCTCCTAT

-1568 CACATTTTGA TTTTCTTATC CCACATTTTT AAAATTTTGT AAGTTACTAT

-1518 ATATTTTTTA AATTATAAAA ACAACTCCAT TTGTGTGGGA TATGTAAATC

-1468 CAAAAAATAG GGGCATTTTA TGTCCATTGA ATATGAGATG CTTACAACCA

-1418 GGGAGTAAAA TTTCGACAAA ATTTTGGTAT TTTTGGGGTC CGATAATTTT

-1368 AGCAGGGTAT TTTTAGAAGT ATATTATTTC GCGAATTTTT GGCCAATTTT

-1318 GGTTAAATTT CAAAATTTGT CAATAATTTC GATATTTTTC GACCAATTTC

-1268 AAAAAAATTT GGCCAATTTT AAAGTTATTT CAATTTTTGT TCAAAATTTC

-1218 GGTATTTTTC GTTGAACTTC TTACTATTTT CTGGTAAAAT TTTTTGGCAG

-1168 CAGTTGGTGT TTTTGTTAAA GTAAAACACA CGTGTATATA TATTAAACAT

-1118 AATATACTTG AAAATAAAAT TCAATTTTTT TTATCAAACA CCGAAATATT

-1068 ATTTTTTAAT GAAAATTTTA AAATTTTGAT AATTTAGAGT CACCAAAATA

-1018 TTACCGAAAT CTAAAATTTT CTTCATTTCC TCCACCAAAG TGTAAGATGT

-968 ATATAACAAT TTTCTAAAAA AAATTGCTTA TGAAACTGAT TCTTTATTTG

-918 AAAAAAAAAA TAAAATTGAC ACTTTCAAAA TGGGTAATGA TTCATGGCCT

-868 CACTGAGGCC ACGGCCTCAT ATGATGTGGC TATAGCCACA TCATTAATTC

-818 TCCTTGTAAT TAATATATTT AATGTTAAAA AAAAAATAAA GTTAATTATA

-768 AAAGGTAAAA AAGAATGTGA AGTCATTATT TCGGATGGCC TCAAAATTTT

-718 AATGAAAAGA GAGGGAGCTC GTGTGATCCA CTTGCCTACC ACAACGAAAC

-668 ATTTTCAATC TTATTGGTAT ATACCCCCAT TTATAGAGAC CACAAATCAA

-618 ATTAGTACCA AAGCATAACA TCCAAACAAC GACAAACCGG TCAAAACGGT

-568 CAACCCCTCA ACCTCGAAAA CTATCAGGCC CAGAAAGCTA TACAACACGT

-518 GCTCTACACC TTCCAGTCAC AGTCTACCAC TACTGAGCGT GTAGTTCACG

-468 TACTATATAA GCAGGTAACT CTTTAGTATA TACTTTATTA GGATGAGCAG

-418 GATACGATTT CGTAGCCTAT CTACTCTAAA GCATCTCGAA TCGTATAAGT

-368 CCGATACGTA TCAGCTGATT TTCTCTTTTA TCTGACCAGC TTGCAGAGGA

-318 GTCCTCTTTA ATCTTTATAT TCGAGTCCAA GATTATTTTC ATGGAAAACT

-268 CGGTCATTTC ATTTTCCTCC GCGCCAAACA CCCAAAAGTC CAAAATTAAA

-218 ATGAAATATT ATTAGTATAA TAGAAGAGAA AATCCTGAAA AAAGGACAGG

-168 CGTGATAATC TTTCTCAACT AAAACCCTCT CTCTCTCTCT CTCTCTCTAT

-118 TTCTACAGTC CCAGAGAGGG AAACCTATCT GGATATGTTT GTGGGTATGA

-68 GATTGAGCTT CTCTGTCTTG TTCATGATAT AATGAGAAGA GGCTTTGGTC

**CAAT-box TATA-box**

-18 TCTTCTTCTC AGAAGAAG**AT G**

**D.The promoter of *PsBG6***

-2002 CTTTTTCTCT CATCATATGT ATAAGGTTTT GTGTTCTAGG GCTTTTTGGT

-1952 TGATAATGTA AGATAATGGG TTGTTATGTA CTAATTTTAT AAACTGTGTA

-1902 CAATACTTGA TTTGCTTATC TCTTGACATA AAGAGTACAG TCTACAGAGT

-1852 AGTGCTGCAT GTGAATAAGT TCATCGTCTT GTTTAATACT TTCGTGCAAA

-1802 CCAGTTAGAT TCATTTCCCC ACCATTAATC TTTTACTAAG TTGCTTAAAT

-1752 AACCTTATGA CTGTATTCAT CTCTAATGCT TTAAGTTTCT TGTAAGGAAA

-1702 TAAAATCTAT GACTTAGTGT AACTAATAAA TAATGCCTTC AAAACTATAG

-1652 CAGAAAAGGT ATATTAGTAA TTCCGACCTA ATAACAACCA AAGTTGGGTT

-1602 GGCTTAATGT AGAAGTTCTC AAGAATTGAT TGCTAGGTAA CTTAATGGGG

-1552 AACCGATGAC CACTTCTAAA CATACCAAAA CACATCTTTC AATGGTCATT

-1502 CGCTGGAATT TGAAATTCCC AAAATCTTTT CCACTATAAG GTTCATTCAT

-1452 TCCGAGTTAA AGATGTGGTA AATTTGTTAA GGAATTCTCA TTGGGGTCAG

-1402 GGCTTAGACT TAGGCTTCTA GCTGGTGATC CTAAACCCTA AATCTTAACC

-1352 TAATTTTGAT GAGGGATTCC AATGCCATGG GAATAAATCA GAGAAAAGAA

-1302 ATGAAATGGT TGGTTTCCTA TTAAGTTACC CACCGGTCGA TTCTTTGAGC

-1252 ATCCTTTGGC TTAATATGTA GTATTGATAC CTCATTCATC CCATTGGAAG

-1202 TTATGGTGCA CTGCCCAAAT GAATGTTATG GTGCACTGTC CATGATAGGT

-1152 TTATAATTGT CGTAATATTA TACAACCTCA TGTTTATCTA ATTCTGATTT

-1102 TGGATCAATA ATTTGACAAG CTTGTTTACT TTGTGCCTCA TTTTGTCTTT

-1052 TGGTTGGTCA TGCAAAATCA TGTCATTTCT TGATTGAAAA CCTGAACCTG

-1002 CAAGACAACT GTATCGATGA TATTAATCTT CCACATTAAC TTTAATCTTA

-952 CTCTTCCCCA AGTATACTCT CAGTGAGAAC TAGGCCACTG AGAGTAACTG

-902 GTGCACTCGA AGGTCTTCTG TGCACATGCC CGAACCATCT TAACCTATTT

-852 TTTTCCCATT TTATCTCTAA TTGGTGATAC TCCAACAGTT TTTAAGATGT

-802 GATCATTTCT AATCACACGC ATTCTAATTT TACCACACAT CCATTTCAGA

-752 ATTCACATTT CCACTTCAAA GGGTTATTTT ATTTCCATTC TGGAATCGAA

-702 GTGGCATTTC TTGTGTAGTT TCTTAGCTGC CCAACTTTCT GTCCCATAAA

-652 ATACAAGTGA TCTAATAGTT GTTTTATACA CTTCATTTCC AGTGTTAACA

-602 GGATTCGTCA TTTTGTTAAT TCTATTACTA ACATCATGTC CTATTTCACC

-552 TTTTTATGCA TAACTGAGCT TATATAACGA AATGTTCACT TTTTGTATCT

-502 CTTGATTCTC AATCTTCACT CAAACCTCAT TTGTATGTCT AGATTACTAA

-452 AGGTAGACTC CGTATTCACT AAAGCTCACT AAAACTGAGC TTATATAACG

-402 AAATGTTCAC TTTTTGTATC TCTTGATTCT CAATCTTCAC TAAAACCTCA

-352 TTTGTATGTC TAGATTTACT AAAGGTAGAC TCCATATTCA GTTTATTTGA

-302 TTACAGTAAT TGGGCTTCTG GTGATATGTT TCAGTTGATT TTTTGCCATA

-252 TTATATTGCT TAGTTAAGTT AGCCAATTTG TGGCATTCAC TGACAAATCT

-202 TTTGAACATA ATAAGCTGAA TATTGTTTAT TTGATGTATA TCGTTGGCCC

-152 ACGAACAGCT TAATCTTTTA GGTGAGTTAG TGAGTTTACA ATTTAACATG

**CAAT-box**

-102 GTGTAAGAGT GGCTATCATG TAAAATTTTG GGTTCATTGC TTTTGTGACG

**TAAT-box**

-52 TACACTCCCA CCTCTTCTCT CCTGGTTGTG GTCTTTTGCC ACTGTTCTTC

-2 TA**ATG**
