# Supplementary material for: Dual functions of PsmiR172b-PsTOE3 module in dormancy release and flowering in tree peony (Paeonia suffruticosa)
Source: Hortic Res. 2023 Feb 21;10(4):uhad033. doi: 10.1093/hr/uhad033 (PMC10120838; doi:10.1093/hr/uhad033)
Supplement: Web_Material_uhad033 [file web_material_uhad033.zip › Supplemental file 4-20220918.docx]

**Supplemental file 4**
