# Supplementary material for: Dual functions of PsmiR172b-PsTOE3 module in dormancy release and flowering in tree peony (Paeonia suffruticosa)
Source: Hortic Res. 2023 Feb 21;10(4):uhad033. doi: 10.1093/hr/uhad033 (PMC10120838; doi:10.1093/hr/uhad033)
Supplement: Web_Material_uhad033 [file web_material_uhad033.zip › Table S1 The primer information used in this study1230.docx]

Table S1 The primer information used in this study

| Name | Sequence (5’-3’) | purpose |
| --- | --- | --- |
| PsAP2GSP5’ | GGAAGCCCAGTACTCTGTCGGCGAA | 5’RACE PCR |
| PsAP2GSP3’ | GAGCGGCCATCAAGTTCCGGGGAGT | 3’RACE PCR |
| PsmiR172a-real | CGCAGAATCTTGATGATGCTGCAT | qRT-PCR |
| PsmiR172b-real | CGCGGAATCTTGATGATGCTGCAT |  |
| PsmiR172d-real | GCGAGAATCCTGATGATGCTGCAG |  |
| Pre-PsmiR172a-real | TCTTGATGATGCTGCATTGGCA |  |
| Pre-PsmiR172b-real | CTACCAATGTCTTGAACGTGAG |  |
| Pre-PsmiR172d-real | CCTGATGATGCTGCACTGGAG |  |
| PsU6 | CGGCGGACTTGAATTTGTGTCATG | internal reference |
| PsAP2-real-F | GCATAAATGTGGAAGATGGGAAGC | qRT-PCR |
| PsAP2-real-R | TGACAGCGTCCTTGCCATTG |  |
| JI447049-real-F | GGTTCTCGAGCCATGACCAA |  |
| JI447049-real-R | GTGGTGCACCCACATGTTTC |  |
| JI458458-real-F | AACCTGCGTTATTGCTCCCT |  |
| JI458458-real-R | TCGAACGTGCCTAGCCATAC |  |
| PsActin-real-F | GAGAGATTCCGTTGCCCTGA |  |
| PsActin-real-R | CTCAGGAGGAGCAACCACC |  |
| PsCYCD-real-F | GAGGCCGTGGATTGGATTCT |  |
| PsCYCD-real-R | AAAAGGGGCACTTGGGTCTC |  |
| PsEBB1-real-F | AATAGCCCGCGAAGTCCAAA |  |
| PsEBB1-real-R | GGGATCTGATGAACCAGCCC |  |
| PsEBB3-real-F | GGTGAGATTACTCCGCCACC |  |
| PsEBB3-real-R | CGACCCTGAATCTGAGACCG |  |
| PsBG6-real-F | CCTACTACCCGGCCACAAAG |  |
| PsBG6-real-R | CTACTGAAAGCACCCGCAGA |  |
| PsAP2-GFP-F | atacaccaaatcgactctagaATGTGGGATCTGAACGATTCGC  ( *Xba*I) | PsAP2 subcellular localization |
| PsAP2-GFP-R | gctcaccatggtaccggatccTGGAGATGGTCTCATGAGAGAGTAGT  (*Bam*HI) |  |
| PsmiR172b-GUS-F | aagaacacgggggactctagaCATCCACAAACTTTACGTTTTAACCA  (*Xba*I) | PsmiR172b:GUS vector |
| PsmiR172b-GUS-R | ggactgaccacccggggatccTTGTTTATTGCCAATGCAGCA  (*Bam*HI) |  |
| PsAP2-GUS-F | gagaacacgggggactctagaATGTGGGATCTGAACGATTCGC  (*Xba*I) | PsAP2:GUS vector |
| PsAP2-GUS-R | ggactgaccacccggggatccTGGAGATGGTCTCATGAGAGAGTAGT  (*Bam*HI) |  |
| mPsAP2-GUS-F | gagaacacgggggactctagaATGTGGGATCTGAACGATTCGC  (*Xba*I) | Mutant PsAP2:GUS vector |
| mPsAP2-GUS-R | ggactgaccacccggggatccTGGAGATGGTCTCATGAGAGAGTAGT  (*Bam*HI) |  |
| PsAP2RLM5’R | ATGGCCATGGACAAAGCATTTCTCCGA | RLM 5’ RACE |
| PsAP2-P1 | GAAACGGCAAGTCGGTGGAGGT | RT-PCR of *PsAP2* |
| PsAP2-P2 | CGCAGGGAAGGATAAAAAGGAAAGA |  |
| PsAP2-P3 | AGCAGAGAAATGGGTTCCACTACT |  |
| PsAP2-P4 | TAGTTGAAGATTTCGTCTGATTGTTAG |  |
| PsAP2-pBI121-F | ccccgggATGTGGGATCTGAACGAT  (*Sma*I) | PsAP2 overexpression vector |
| PsAP2-pBI121-R | cgagctcTATGGAGATGGTCTCATGAGAGAG  (*Sac*I) |  |
| *PsmiR172b*-pBI121-F | tcccccgggTGAGTTTGAGTTTGAGAG  (*Sma* I) | PsmiR172b overexpression vector |
| *PsmiR172b*-pBI121-R | ggactagtTACTTCAAGTAGAAACAAACCTACCTG  (*Spe* I) |  |
| PsEBB1F-pHis-F | gactcactatagggcgaattcGGGATGTAAGCCAAATGCTCTC  (*EcoR*I) | Yeast vector of F fragment of *PsEBB1* promoter |
| PsEBB1F-pHis-R | attactagtggatccacgcgtCATATTCTCTGTCTCCTCTCTCACTCTT (*Mlu*I) |  |
| PsEBB1F1-pHis-F | gactcactatagggcgaattcCTTTGGACTTTTGCAACCGACC  (*EcoR*I) | Yeast vector of F1 fragment of *PsEBB1*promoter |
| PsEBB1F1-pHis-R | attactagtggatccacgcgtCATATTCTCTGTCTCCTCTCTCACTCTT  (*Mlu*I) |  |
| PsEBB1F2-pHis-F | gactcactatagggcgaattcGGGATGTAAGCCAAATGCTCTC  (*Eco*RI) | Yeast vector of F2 fragment of *PsEBB1* promoter |
| PsEBB1F2-pHis-R | attactagtggatccacgcgtCAGTCACGTGATAGCAAATGCTC  (*Mlu*I) |  |
| PsAP2-AD-F | gtaccagattacgctcatatgATGTGGGATCTGAACGATTCGC  (*Nde*I) | Yeast vector of PsAP2 |
| PsAP2-AD-R | atgcccacccgggtggaattcTGGAGATGGTCTCATGAGAGAGTAGT  (*Eco*RI) |  |
| PsEBB1-C-repeat-F | AACCGACACCGACACCGACACCGAC | C-repeat probe of *PsEBB1* synthesis |
| PsEBB1-C-repeat-R | TTGGCTGTGGCTGTGGCTGTGGCTG |  |
| PsEBB1-mC-repeat-F | AAGCGACAGCGACAGCGACAGCGAC | Mutant C-repeat probe of *PsEBB1* synthesis |
| PsEBB1-mC-repeat-R | TTCGCTGTCGCTGTCGCTGTCGCTG |  |
| MBP-PsAP2-F | gagggaaggatttcacatatgATGTGGGATCTGAACGATTCGC  (*Nde*I) | Fusion vector of PsAP2 with MBP |
| MBP-PsAP2-R | TtaattacctgcagggaattcTTATGGAGATGGTCTCATGAGAGAGT  (*Eco*RI) |  |
| AtSOC1-real-F | TCGCCAGCTCCAATATGCAA | qRT-PCR |
| AtSOC1-real-R | TCTGTTGCAGCTCCTCGATT |  |
| AtLFY-real-F | ATTGCTAAAGACCGTGGCGA |  |
| AtLFY-real-R | TGTAACAAGCCTGACGCCAT |  |
| AtActin-F | GACCTATACCAAGCCGAAG |  |
| AtActin-R | CGTTCCAGCACCACAATC |  |
| [AtCYCD3;1-F](https://www.arabidopsis.org/servlets/TairObject?type=gene&id=129262) | AACAAATGCCACCGTCTCCT |  |
| [AtCYCD3;1-R](https://www.arabidopsis.org/servlets/TairObject?type=gene&id=129262) | TCAATCACGCAGCTTGGACT |  |
