# Supplementary figures and images for: Dual functions of PsmiR172b-PsTOE3 module in dormancy release and flowering in tree peony (Paeonia suffruticosa)
Source: Hortic Res. 2023 Feb 21;10(4):uhad033. doi: 10.1093/hr/uhad033 (PMC10120838; doi:10.1093/hr/uhad033)

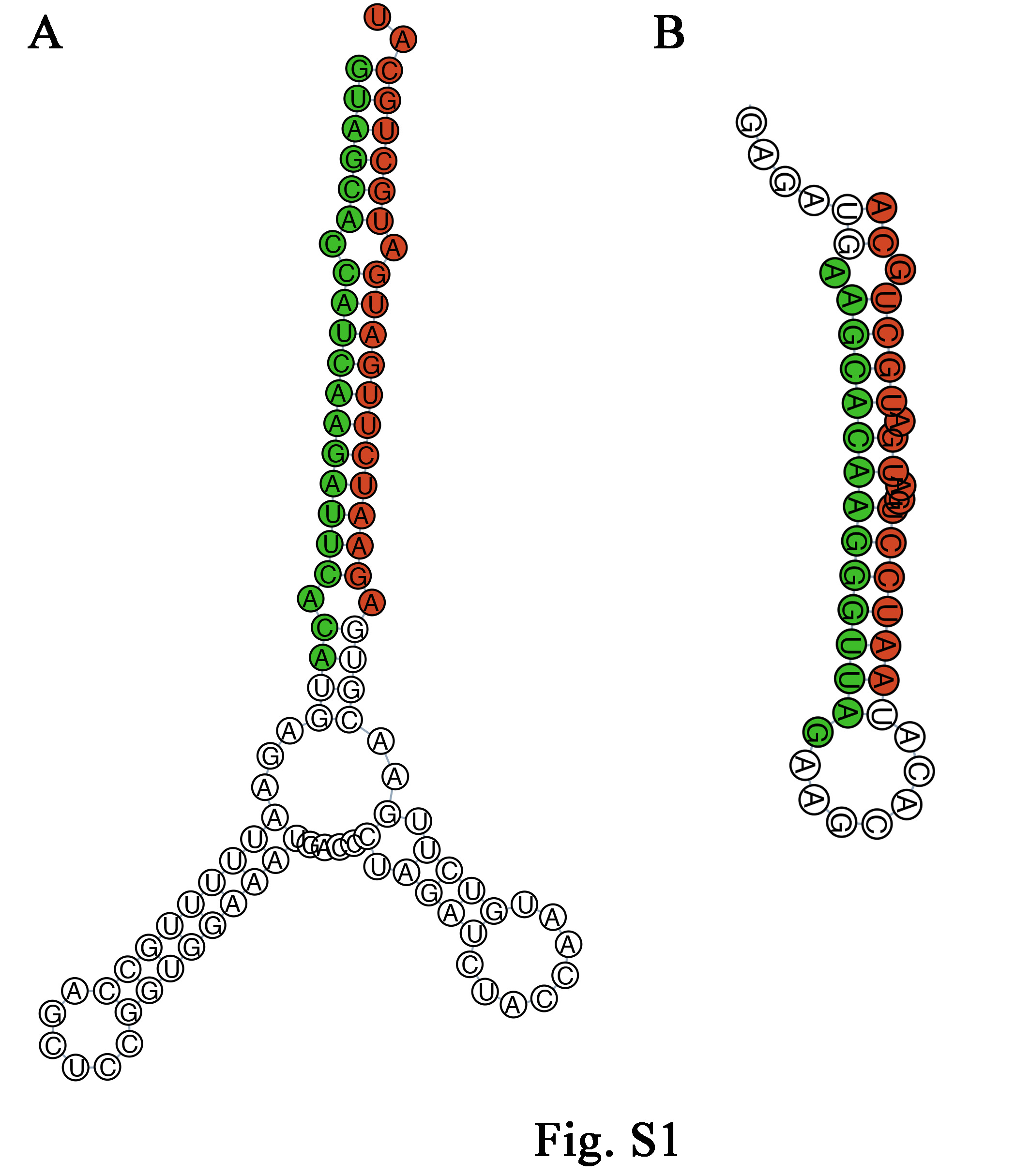

Supplement: Web_Material_uhad033 [file web_material_uhad033.zip › Fig S1__.jpg]

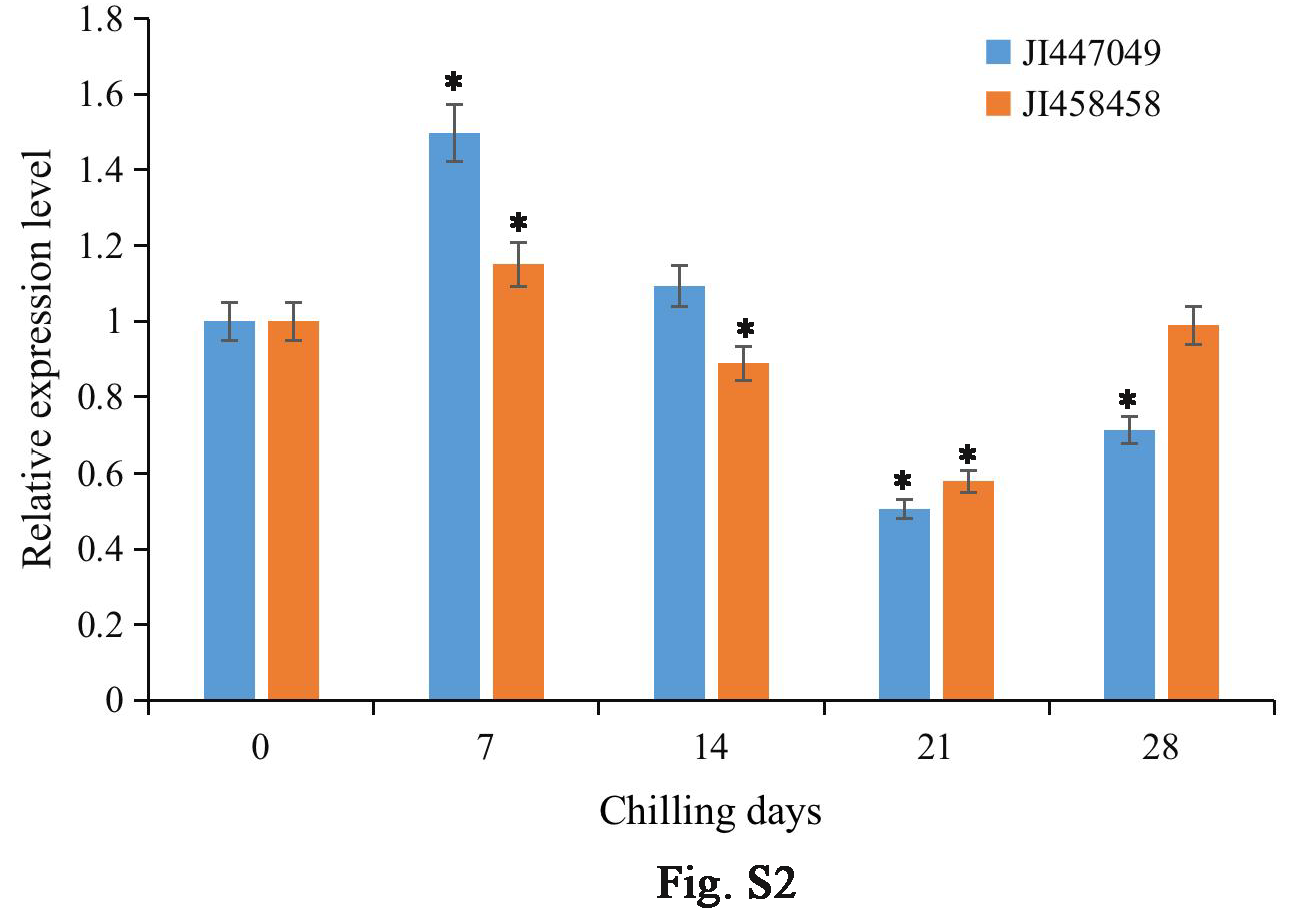

Supplement: Web_Material_uhad033 [file web_material_uhad033.zip › Fig. S2.tif]

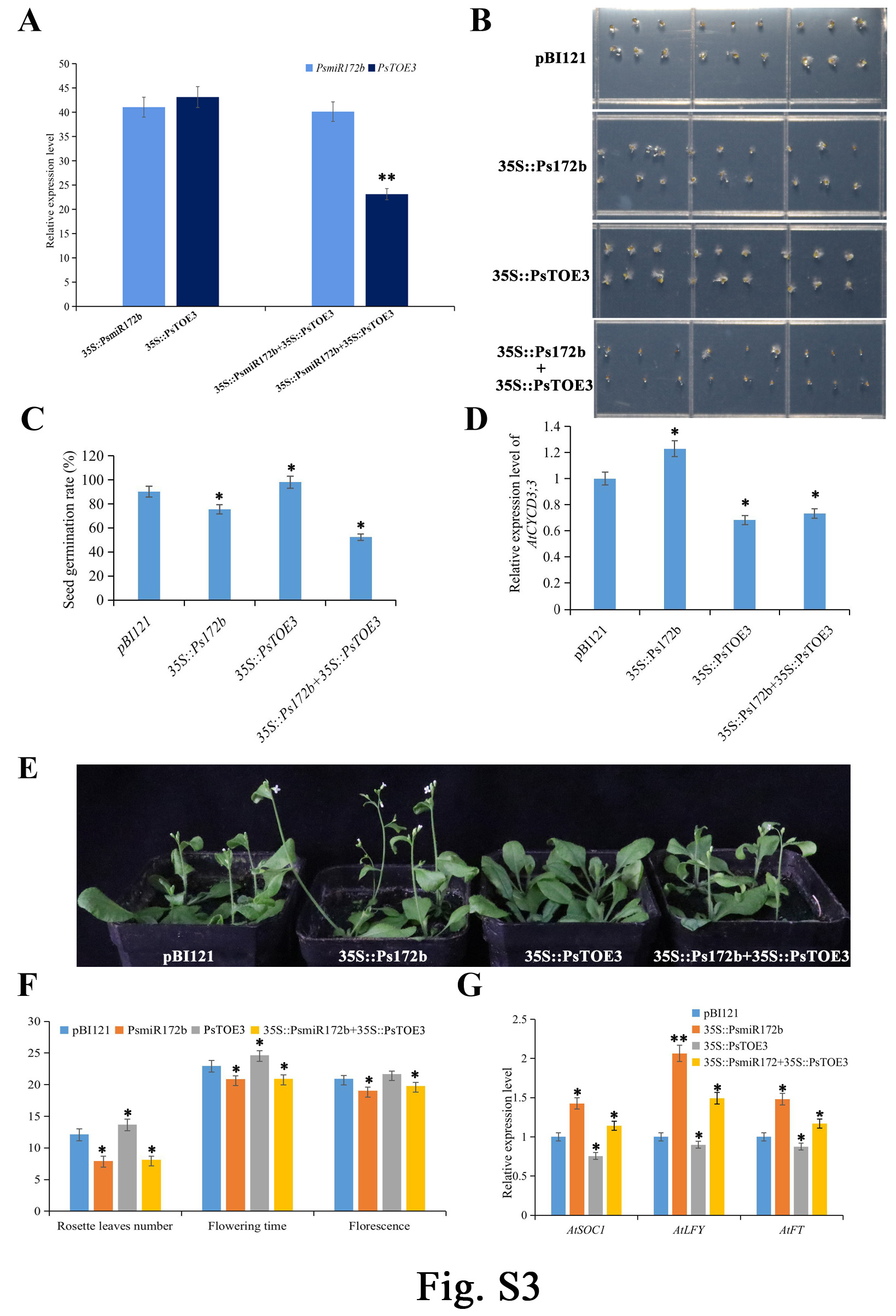

Supplement: Web_Material_uhad033 [file web_material_uhad033.zip › Fig. S3__.jpg]

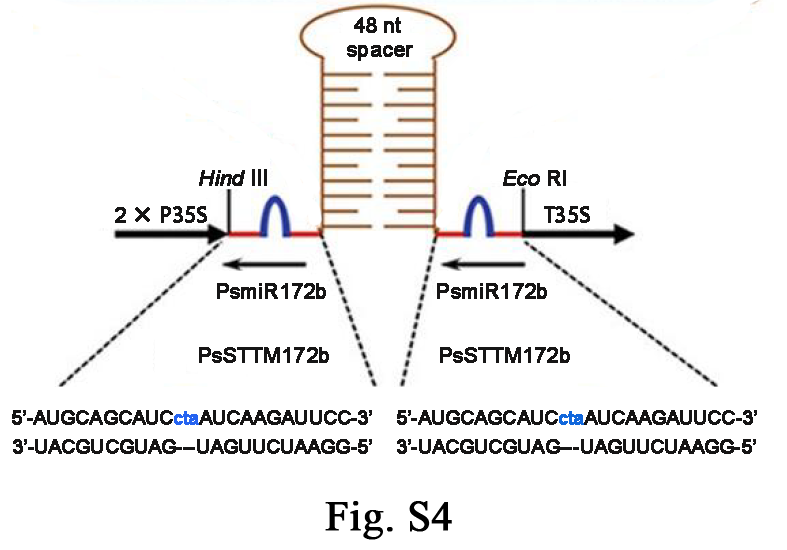

Supplement: Web_Material_uhad033 [file web_material_uhad033.zip › Fig. S4-STTM172.tif]
